# A Review about the Recent Advances in Selected NonThermal Plasma Assisted Solid–Gas Phase Chemical Processes

**DOI:** 10.3390/nano10081596

**Published:** 2020-08-14

**Authors:** Vincenzo Palma, Marta Cortese, Simona Renda, Concetta Ruocco, Marco Martino, Eugenio Meloni

**Affiliations:** Department of Industrial Engineering, University of Salerno, Via Giovanni Paolo II 132, 84084 Fisciano (SA), Italy; vpalma@unisa.it (V.P.); mcortese@unisa.it (M.C.); srenda@unisa.it (S.R.); cruocco@unisa.it (C.R.); mamartino@unisa.it (M.M.)

**Keywords:** nonthermal plasma, plasma chemical process, packed bed reactors: DeNO_x_, volatile organic compounds decomposition, catalysts, process intensification, soot abatement

## Abstract

Plasma science has attracted the interest of researchers in various disciplines since the 1990s. This continuously evolving field has spawned investigations into several applications, including industrial sterilization, pollution control, polymer science, food safety and biomedicine. nonthermal plasma (NTP) can promote the occurrence of chemical reactions in a lower operating temperature range, condition in which, in a conventional process, a catalyst is generally not active. The aim, when using NTP, is to selectively transfer electrical energy to the electrons, generating free radicals through collisions and promoting the desired chemical changes without spending energy in heating the system. Therefore, NTP can be used in various fields, such as NO_x_ removal from exhaust gases, soot removal from diesel engine exhaust, volatile organic compound (VOC) decomposition, industrial applications, such as ammonia production or methanation reaction (Sabatier reaction). The combination of NTP technology with catalysts is a promising option to improve selectivity and efficiency in some chemical processes. In this review, recent advances in selected nonthermal plasma assisted solid–gas processes are introduced, and the attention was mainly focused on the use of the dielectric barrier discharge (DBD) reactors.

## 1. Introduction

The term plasma, first introduced by Langmuir and Tonks in 1929 to describe the inner region of a glowing ionized gas phase produced by means of an electric discharge in a tube, represents the “fourth state of matter”, consisting in an “ionized gas” into which sufficient energy is provided to free electrons from atoms or molecules and to allow the coexistence of species, ions and electrons [1]. The plasma ionization degree is the proportion of atoms that have lost (or gained) electrons. Plasma is divided into thermal or hot plasma and non-thermal or cold plasma, and the main features of the different plasmas are shown in Table 1 [2].

In thermal plasma, also named equilibrium plasma since the temperature of charged particles is very close to the one of the background gas, a particular environment different from the conventional chemical engineering processes can be provided, in which temperatures of 10^3^–10^4^ K are reached. In nonthermal plasma (NTP), the generated electric field transmits energy to the gas electrons and then energy will be transferred to the neutral species by collisions. In such a way the formation of highly reactive short-lived species occurs, such as O· (Oxygen radical), O_3_ (Ozone), N· (Nitrogen radical), N* (excited Nitrogen radical), N_2_^+^ (positive ions of nitrogen) and ·OH, so promoting the desired chemical reactions [3]. These reactions can be accomplished with just a fraction of the energy that is required in the thermal plasma system. In NTP, electrons have a kinetic energy higher than the energy corresponding to the random motion of the background gas molecules, generally in the range 10^4^–10^5^ °C (2–3 order of magnitude greater than the background gas). One example of nonthermal plasma is the gas filling a fluorescent tube, in which the gas temperature is only around 40 °C, while the temperature of free electrons in the system exceeds 10^4^ °C.

NTP can be generated in several ways, including electrical corona discharges, radio frequency (RF) discharges, microwave discharges, dielectric barrier discharges and electron beams [2]. The choice of the proper reactor design for a specific application is a big challenge, since it requires the optimization of lot of parameters, such as the discharge modes, the operating pressure, the presence of a dielectric barrier or catalyst, the geometry, the polarity, the voltage level; sometimes, the tests will lead to negative results. However, in general, the relatively low temperature of nonthermal plasma allows its combination with catalytic processes, since it can improve catalyst selectivity and, in case of pollutant abatement, the removal efficiency. The chemical potential of NTP has been studied with regard to various applications, such as volatile organic compound (VOC) decomposition, NO_x_ and SO_x_ removal, ozone generation, surface treatments, H_2_ formation, fuel reforming and biomedical use [3], as well as in the preparation of catalytic materials [4] and catalyst regeneration [5]. The main used reactors are briefly described in the following subsections.

### 1.1. Electron Beam

In this kind of reactors, an electron beam, which is formed in a separate generator, such as a cathode tube, is injected into the exhaust gas. The energy of the electrons is absorbed by the components of the gaseous mixture proportionally to their mass fraction. The energy of electrons can be much higher in the e-beam reactor than in other reactors. Disadvantages of the e-beam reactor include the need for a special reactor for generating the electrons and poor efficiency in transferring the electrons into the exhaust gas, as well as the requirement for high/ultra-high vacuum compatibility of all parts of the process [1].

### 1.2. Corona Discharges

These reactors are characterized by the formation of a nonuniform electric field between two concentric electrodes where their radius of the curvature is the smallest. In this way the realized discharge mode is the streamer one and the ionization zone is spread over the entire gap, so allowing the possibility to set the discharge gap as large as 10 cm or more, which is highly appropriate for large scale application. Important features of these reactors are (i) the absence of a dielectric for the plasma generation and (ii) the use of pulsed electric field to prevent plasma from going into the thermal mode and forming an arc. In the case of some environmental applications, such as decomposition of CCl_4_, pulsed corona reactors resulted extremely less energy efficient than the electron beam processing, but, as mentioned before, the drawbacks of the latter in terms of requirement of a separate reactor and difficulty of electron generation made its application limited [1].

### 1.3. Dielectric Barrier Discharge

**Figure 1:**
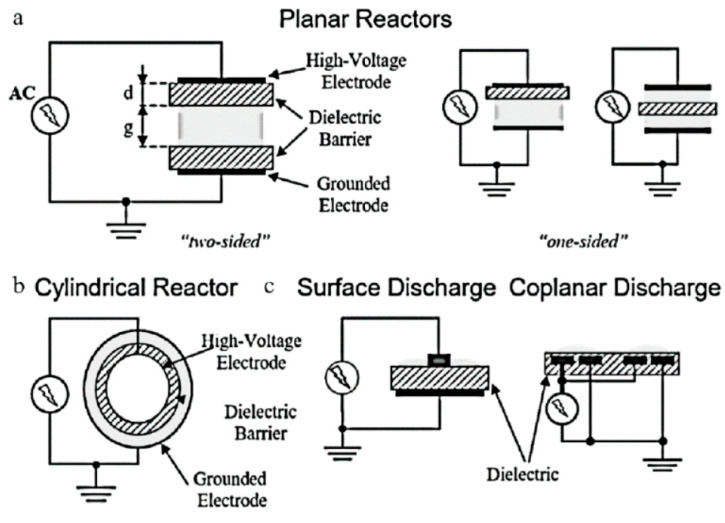
Some typical dielectric barrier discharge (DBD) reactor configurations. (**a**) Planar; (**b**) cylindrical; (**c**) two discharge types of surface discharge and coplanar [6].

Dielectric barrier discharge (DBD) plasma is one of the most common methods for NTP generation. A DBD reactor is basically composed by a set of electrodes with at least one dielectric barrier between them; different typologies of DBD reactors are shown in Figure 1 [6].

Since a dielectric barrier is present, higher voltage are required because a high electric field between the electrodes is necessary for plasma formation and so causing breakdown in the gas. Usually the dielectric barriers are in quartz glass, silica glass or alumina; other used materials are ceramic materials or polymer layers in special cases. Differently from the corona discharge reactors, the DBD ones produce a homogenous discharge with low energy consumption, so resulting in no spark formation inside the streamer channels, overheating and local shock waves. Additional advantages of the DBD reactors are their scalability, effectiveness, and low operational cost. These advantages make these reactors feasible for pollutant abatement, such as CO, NO_x_ and VOCs, as well as for ozone generation [1]. However, since the DBD reactors vary in geometric configuration and operating parameters (discharge characteristics depend on the gas composition, type of dielectric material and operating conditions of voltage and frequency), the comparison of their performance in terms of discharge power characteristics is really difficult [7,8].

### 1.4. Dielectric Packed Bed Reactor

**Figure 2:**
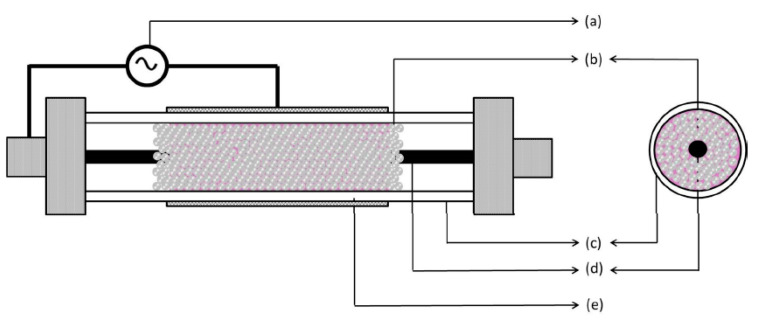
Schematic diagram of a packed-bed nonthermal plasma (NTP) reactor and its cross-sectional view (**a**) Power source; (**b**) packing material in discharge gap; (**c**) dielectric barrier; (**d**) high voltage (or powered electrode) and (**e**) ground electrode [9].

The dielectric packed bed reactor is like the DBD, but in the former pellets of dielectric materials are in the gap between the barrier and the electrode (Figure 2) [9].

The presence of the pellets allows the use of low applied voltages can be used for the plasma generation over a relatively large separation of the electrodes; in fact, when an external electric field is applied, the pellets spontaneously polarize in its direction, resulting in a high electric field at their contact points. Furthermore, the pellets can be catalyzed, in order to obtain a synergistic effect between plasma and catalysis for the improvement of the process efficiency. However, the presence of pellets has some disadvantages, mainly consisting in the high pressure drops and attrition of the pellets [1].

### 1.5. Surface Plasma Discharge

In this reactor, one side of the dielectric barrier is covered entirely by one of the electrodes and only partially by the other one, so allowing the distinction from the DBD reactors. The plasma is generated next to this dielectric surface, which is in contact with the gas and the surface plasma covers the entire dielectric surface. A feature of this discharge is that after a few nanoseconds, charge begins to build up at the dielectric surface, which has the effect of reducing the electric fields outside the dielectric, eventually extinguishing the discharge [1].

In this review, the recent advances in selected nonthermal plasma assisted solid–gas processes are presented. The attention is focused on the pollutant abatement and on the intensification of some processes, so the studies regarding the use of NTP in the abatement of SO_2_ (Section 3), H_2_S (Section 4), NO_x_ (Section 5), soot (Section 6), VOC (Section 8), as well as the ones on the ammonia production (Section 2) and the CO_2_ utilization (Section 7) are reviewed in the following pages.

## 2. Ammonia Production via NTP Technology

**Figure 3:**
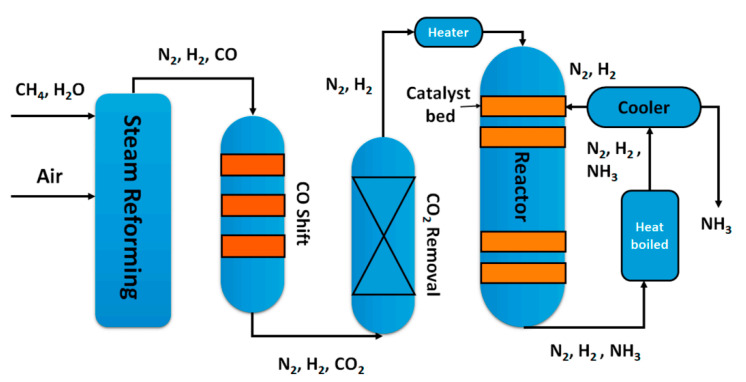
Flow scheme of the Haber–Bosch process [11].

Ammonia is one of the most important chemicals, involved in several industrial processes, including production of fertilizers, explosives, polymers and nitric acid. The actually used production process, the Haber−Bosch process (Figure 3), is the result of a series of patents published in the early twentieth century [10]. The conventional Haber–Bosch process is based on the exothermic equilibrium reaction (1) between nitrogen and hydrogen [11], it is catalyzed, it is typically carried out at 500 °C at a pressure of 150–200 bar, so requiring huge energy supply [12].

N_2_ + 3H_2_ = 2NH_3_  ΔH = −92.2 kJ mol^−1^ (1)

Although it is an exothermic reaction, it is conducted at high temperatures because of the slow nitrogen triple bond cleavage and to avoid the poisoning of the catalysts by the adsorbed nitrogen; however, the high pressures are used to balance the detrimental effect of the temperature [13].

It is therefore clear that a process with these characteristics is not very sustainable and technically unusable for localized applications [14] in the ammonia distributed production. A promising alternative is the “plasma-assisted nitrogen fixation” process [15], which can be considered eco-friendly, due to the no greenhouse gas emissions and waste production and suitable for small-scale and distributed production. On the other hand, the energy efficiency of this process is far from being optimized, so that the combination with renewable energies seems to be, at the moment, the only viable route [16].

It has been shown that the ammonia synthesis can be realized without the use of catalysts, by using water vapor as a hydrogen source for nitrogen fixation into NH_3_, by non-equilibrium plasma. High NH_3_ selectivity (up to 96%) and an increase in production rate, compared to N_2_ dry in contact with liquid H_2_O (up to 0.064 mg h^−1^), with low quantities of vapor saturation of the N_2_ feed gas, were reported [17]. However, with higher H_2_O vapor contents, the selectivity was lower (~60%−85%), but the combined yield to all N_2_ fixation products (i.e., NH_3_, NO_3_^−^, NO_2_^−^) increased. The total N_2_ fixation product yields increased when nitrogen was replaced with air, but the selectivity to NH_3_ was drastically depressed. The energy consumption was around 100 MJ mol^−1^ for NH_3_ or 15 MJ mol^−1^ for total N_2_ fixation, which were in the reported range values for plasma assisted catalytic NH_3_ production.

On the other hand, most of the works published in recent years are based on the use of catalysts, in order to make the process that uses plasma more competitive than conventional methods. In this section, the results reported in selected recent published papers, on the plasma-assisted ammonia production in presence of catalysts, will be reviewed, moreover, at the end of the section a summary table is provided, on the efficiency of selected catalytic formulations (Table 2).

### 2.1. Mechanisms

**Figure 4:**
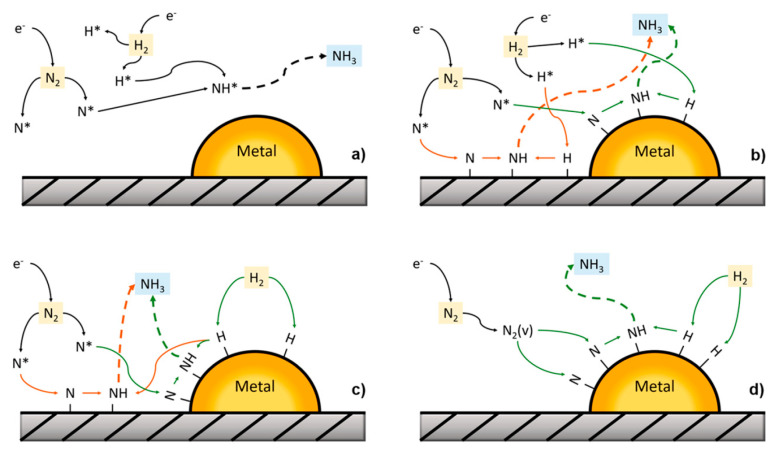
Suggested reaction mechanisms of plasma-phase ammonia synthesis. (**a**) plasma-phase ammonia synthesis; (**b**) Surface-enhanced plasma-driven ammonia synthesis; (**c**) plasma-enhanced semicatalytic ammonia synthesis; (**d**) plasma-enhanced catalytic ammonia synthesis [18]. Reproduced with permission from Kevin H. R. Rouwenhorst, Hyun-Ha Kim, and Leon Lefferts, ACS Sustainable Chem. Eng.; published by American Chemical Society, 2019.

Four different types of pathways [18] for ammonia synthesis in the presence of plasma were suggested: (a) plasma-phase ammonia synthesis, (b) surface-enhanced plasma-driven ammonia synthesis, (c) plasma-enhanced semicatalytic ammonia synthesis and (d) plasma-enhanced catalytic ammonia synthesis (Figure 4). In the plasma-phase ammonia route the N* radicals and H* radicals recombine in the plasma environment to produce ammonia, while in the surface-enhanced plasma-driven ammonia synthesis the N* and H* radicals adsorb on the catalytic surface, followed by NH_x_ species hydrogenation and ammonia formation, which may occur on the surface or in the plasma–environment [12]. These two mechanisms are essentially diffusion limited [18] and shown a low apparent activation energy smaller, typically lower than five kilojoules per mole. In the plasma-enhanced semicatalytic ammonia synthesis route, the N* radicals adsorb on the surface and the H_2_ is dissociated over the catalyst [19,20]. Finally, in the plasma-enhanced catalytic ammonia synthesis both H_2_ and N_2_ adsorb with a dissociative mechanism on the catalyst surface, subsequent NH_x_ surface hydrogenation and ammonia desorption occur over a transition metal [21].

Van Helden et al. [22] studied the ·NH and ·NH_2_ radicals production mechanisms, both in expanding N_2_–H_2_ plasma and in expanding N_2_ plasma with the hydrogen added in the background, by measuring their density evolution along the expansion axis with cavity ring-down spectroscopy. For the experimental setup was used a cascaded arc source channel with a diameter of four millimeters and a length of 30 mm, realized with five stacked water-cooled insulated copper plates, and the applied power was around five kilowatts. The measurements shown an ·NH radical density of 5 × 10^18^ m^−3^ in both the plasma cases and up to 7 × 10^18^ m^−3^ for the ·NH_2_ radical in the case of expanding N_2_–H_2_ plasmas. Moreover, in the N_2_ plasma with hydrogen injected in the background, at z = 10 cm, the 30% of the radical flux is ·NH, while in the N_2_–H_2_ plasma, at z = 10 cm, the ·NH and ·NH_2_ radicals are about the 60% of the radical flux, thus concluding that ·NH_x_ radicals play a crucial role as precursors for the N and H atoms, key steps in the surface production of N_2_, H_2_ and NH_3_.

### 2.2. Ru-Based Catalysts

Ruthenium-based are the most studied catalysts for the plasma assisted ammonia synthesis. Peng et al. [23] investigated a Ru-based multifunctional catalytic system deposited on mesoporous Si–MCM-41 for the non-thermal plasma synthesis of ammonia. The results shown that the synthesis efficiency increased with the frequency values >22,000 Hz, decreased with the applied voltage from 5 to 7 kV, while the optimal N_2_ feed concentration was 0.5. The highest ammonia synthesis efficiency achieved was 1.7 g kWh^−1^ at 5000 V and 26,000 Hz. A two-step process was also proposed to minimize the plasma shielding effects, in which the plasma ionization and catalytic synthesis are separated. The effects of operational parameters, in presence of 10 wt% Ru supported on MgO, carbon nanotube (multi-walled), MCM-41 and activated carbon (mesoporous) and Cs, K and Ba as promoters, were also investigated [24]. The results shown an increase of the ammonia synthesis efficiency with the gas flow rate below four liters per minute and substantial plateau for higher flow rates. The power frequency had no effect on the efficiency in the range 8000–16,000 Hz, while a gradual decrease of the efficiency was found from 5000 to 9000 V. Finally, the best performance was obtained with Ru catalyst on carbon nanotube support and as Cs promoter, while the optimal conditions resulted in a frequency and an applied voltage of 10,000 Hz and 6000 V, with N_2_:H_2_ feed ratio of 3:1, in this condition the efficiency was of 2.3 g NH_3_ kWh^−1^.

Kim et al. [25] investigated the use of Ru-based catalysts, in atmospheric-pressure nonthermal plasma to synthesize ammonia, moreover the effect a promoter such as Mg, K and Cs was also investigated. The results shown that the NH_3_ formation the plasma onset voltage to a lower value, due to the low ionization potential. The NH_3_ formation increased with temperature from 200 °C to 300 °C; below 250 °C the NH_3_ formation was found to be a linear function of the specific energy input (SEI), however, the slope of the correlation function was related to the type of catalyst and power supply. The optimal N_2_:H_2_ feed ratio was found to be 4:1, while the highest ammonia yield was obtained with Ru(2)–Mg(5)/γ-Al_2_O_3_ catalyst at 250 °C, where pulse energization was found to be four times more efficient than AC energization. The addition of small amount of oxygen demonstrated the possibility to regenerate in situ the deactivated catalyst. In further studies [18], the synergy between the catalyst and the plasma was experimentally observed. The apparent activation energy for thermal-catalytic ammonia synthesis is typically located in the range 60–115 kJ mol^−1^, while the calculated apparent activation energy for plasma-enhanced catalytic ammonia synthesis were in the range 20–40 kJ mol^−1^, consistent with the hypothesis that ammonia synthesis was enhanced via plasma-induced vibrational excitations of N_2_, without affecting the hydrogenation steps of NH_x_ species as well as the ammonia desorption. The effects of promoters and supports on activity were similar for both thermal catalysis and plasma-enhanced catalysis, due to the enhanced dissociation of N_2_, suggesting that the N−N bond breaking was still relevant in plasma-enhanced catalytic ammonia synthesis.

### 2.3. Ni-Based Catalysts

Nickel-based catalysts have also been extensively studied. Shah et al. [26] compared the activity of pure Ni metal with that of Ni–MOF catalyst in plasma assisted ammonia synthesis. The Ni–MOF catalyst shown superior catalytic activity that was attributed to the catalyst porous structure, which improves the mass transfer during the reaction, to the presence of open Ni metal sites and to the lower surface hydrogen recombination. Moreover, the Ni–MOF catalyst was stable in the power range 100−200 W, while higher power caused an internal stress, which led to the amorphization of the porous framework, compromising the catalyst performance.

**Figure 5:**
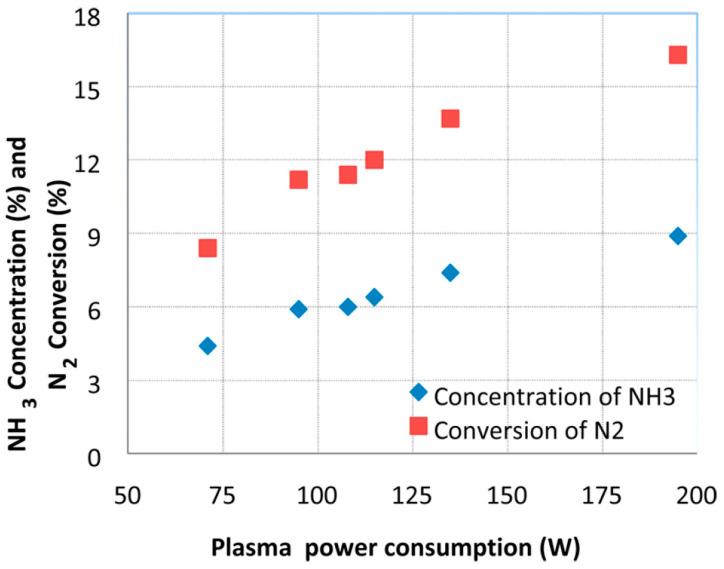
Nitrogen conversion and ammonia concentration as function of the plasma power consumption. Process conditions: H_2_/N_2_ = 3/1, flow rate = 25 mL min^−1^, wall power = 87 W, wall temperature = 130 °C [27]. Adapted with permission from Galip Akay, Kui Zhang, Industrial & Engineering Chemistry Research; published by American Chemical Society, 2017.

Akay and Zhang [27] investigated co-assembled microporous silica supported nickel catalysts in nonthermal plasma reactor, operating at 140 °C and ambient pressure, for the ammonia synthesis. The results shown that the nitrogen conversion reached as well as the ammonia concentration were similar to that achievable by the current industrial process, however, carried out at 100−250 bar and the temperatures 350−550 °C (Figure 5).

Moreover, a stability test, carried out in 72 h on steam, highlighted the excellent performance, as no significant deactivation was found. The XRD diffractograms shown no changes in the catalyst structure after the reaction; however, the crystallite size reduces from three nanometers to less than one nanometer after the reaction. A mechanism for the ammonia synthesis combined to catalytic DBD plasma was also proposed, in which highly energetic electrons dissociate and excite H_2_ and N_2_. Due to the dissociation energy (9.8 eV), the nitrogen activation is the key step, the ·NH radicals are produced by reaction of N* with H* or H_2_, then react with H* or H_2_ to form ·NH_2_ radicals, finally a further reaction with H* or H_2_ gives NH_3_.

### 2.4. Other Catalysts

De Castro et al. [28] investigated the effect of some process parameters on the ammonia synthesis in N_2_/H_2_ direct current glow discharge plasma, such as the nitrogen concentration (in the range 1.5%–33%), the wall materials (tungsten, stainless steel and aluminum as a proxy for beryllium) and the surface temperature (in the range 100–350 °C). The results shown that the ammonia yield increased slowly with the temperature, moreover on the aluminum wall almost the 100% of cracked nitrogen was converted in ammonia at 350 °C. On tungsten and stainless-steel walls, a lower nitrogen concentration increased the cracking efficiency, while only on stainless steel and aluminum an intrinsic dependence on the plasma current was found. The amount of implanted N was related to the ammonia yield, suggesting a competition between N implantation and N/H–N/N recombination on the walls.

Gómez-Ramírez et al. [29] investigated the parameters affecting the ammonia synthesis in a PZT (lead zirconate titanate) ferroelectric packed bed dielectric barrier discharge reactor (DBD), including the operating frequency, the ferroelectric pellets size and the interelectrode distance. The experimental apparatus consisted of a stainless-steel chamber with a stainless-steel plate of 7.5 cm of diameter as active electrode, the operating frequency was in the range 500–5000 Hz with applied voltage 2.5 ± 0.3 kV and 5.5 ± 0.3 kV. The nitrogen conversion increased almost linearly with the frequency because of the changes in the intrinsic electrical properties of the reactor, which can be tuned by modifying the interelectrode spacing and the pellets size. The higher conversion was obtained by feeding a mixture N_2_/H_2_ with a ratio equal to 1/3, at a frequency of 5000 Hz, at a residence time of 60 s. This result was attributed to the increase in the electrical field at the necks between the pellets, where the occurrence of hot spots increased the local temperature.

Hong et al. [30] investigated the ammonia synthesis in a non-equilibrium atmospheric pressure plasma, by using functionalized-nanodiamond and diamond-like-carbon coatings on α-Al_2_O_3_ spheres (DLC-coated Al_2_O_3_). The oxygenated nanodiamond coating improved the ammonia yield of 30% with respect the bare alumina catalyst, while the hydrogenated nanodiamond coating reduced ammonia yield. This result was attributed to different surface reactions, hypothesizing that the presence of the carbonyl bond was related to the ammonia production.

Shah et al. [31] investigated the use of zeolite 5A in the ammonia plasma-assisted synthesis. The formation of microdischarges and a change of the voltage−current characteristics of the reactor was promoted by the presence of the zeolite. The zeolite surface electronic properties were modified by the atmospheric plasma, leading to an enhanced reactive state at the zeolite surface, which promoted the nitrogen. The results shown that the ammonia yield increased with the N_2_/H_2_ ratio, while the ammonia synthesis rate found a maximum for a N_2_/H_2_ equal to 1. Moreover, the ammonia yield decreased with the total flow rate, while the same activity was found after two cycles.

### 2.5. Comparative Studies

Wang et al. [32] investigated the ammonia synthesis in a coaxial dielectric barrier discharge plasma reactor, using water as a ground electrode and M/Al_2_O_3_ (M = Fe, Ni, Cu) as catalysts at ∼35 °C and atmospheric pressure. The average particle size was below 16 nm for all catalysts. Compared to the plasma synthesis without catalyst, the use of a catalyst enhanced the reaction rate and the energy efficiency, the efficiency order was Ni/Al_2_O_3_ > Cu/Al_2_O_3_ > Fe/Al_2_O_3_ > Al_2_O_3_, while the highest rate (471 μmol g^−1^ h^−1^) was obtained by using Ni/Al_2_O_3_. All the catalysts were stable at least for 6 h and the efficiency of the Ni/Al_2_O_3_ catalyst remained unchanged when recycled five times. The operando XPS analysis detected the NH_x_ (x = 0, 1, 2 and 3) adsorbed species on the surface of the spent catalysts, suggesting they were reaction intermediates; the metal sites and the weak acid sites could actually enhance the ammonia production via NH_2_ formation.

**Figure 6:**
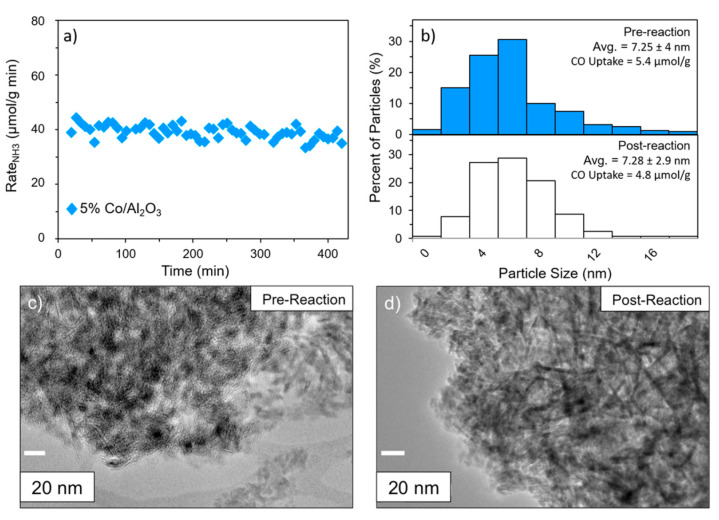
(**a**) Time on stream test over 5% Co/Al_2_O_3_, T = 200 °C, power = 10 W, flow rate 50 Sccm, N_2_/H_2_ = 3; (**b**) particle size distribution before and after the test; (**c**) TEM image before the test; (d) TEM image after the test [33]. Adapted with permission from Patrick Barboun, Prateek Mehta, Francisco A. Herrera, et al., ACS Sustainable Chemistry & Engineering; published by American Chemical Society, 2019.

Barboun et al. [33] investigated the effect of gas composition, temperature and discharge power, on the plasma-assisted ammonia synthesis, by using three different γ-alumina-supported transition metal-based (Ru, Co and Ni) catalysts. The experiments were performed by decoupling the plasma-phase and plasma–catalytic ammonia production rates, thus by using differential reactor conditions and measuring appropriate background reactions, the contributions of plasma–catalytic reactions relative to the homogeneous plasma-phase reactions was evaluated. The results shown that the overall reaction and the plasma-phase reaction rates were first-order both for N_2_ and H_2_, while for the plasma–catalyst interactions were first-order for N_2_ and zero order for H_2_. Both the increase in bulk gas temperature and in power deposition improved the ammonia production, however the discharge power was more effective in in controlling the catalytic active than the gas temperature. A stability test was also performed on 5% Co/Al_2_O_3_ catalyst, showing no significant deactivation in 7 h of reaction time, moreover the comparison of TEM images and particle size distribution between the fresh and the exhausted catalyst shown no obvious change (Figure 6).

**Figure 7:**
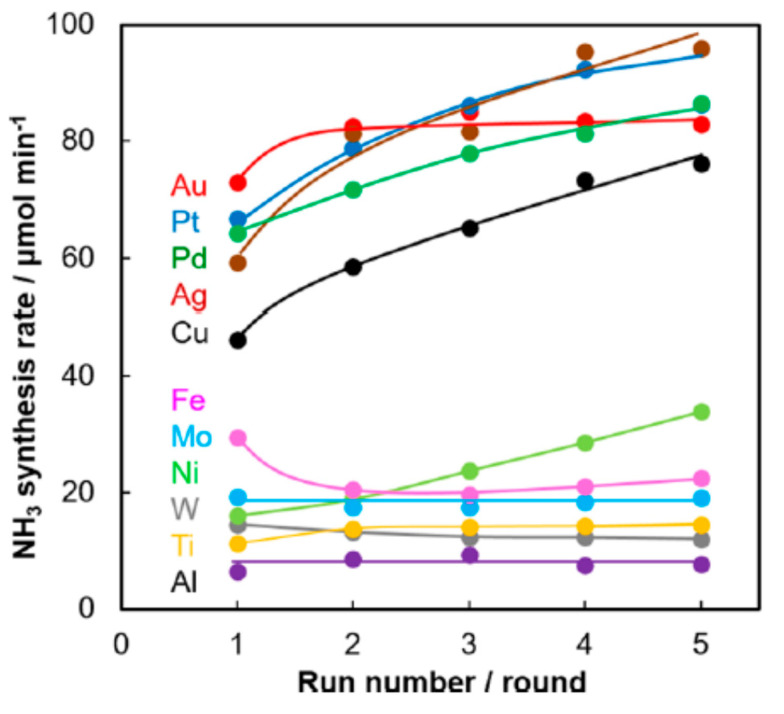
Catalytic activity of the metal-wool electrodes as function of the run number. Reaction conditions: applied voltage = 5 kV; frequency 50 kHz; electrode length = 150 mm; wool-like metal = 61.3 cm^2^; flow rate = 100 mL min^−1^; H_2_/N_2_ = 1 [34]. Adapted with permission from Masakazu Iwamoto, Mao Akiyama, Keigo Aihara, et al., ACS Catalysis; published by American Chemical Society, 2017.

Iawamoto et al. [34] developed wool-like metal electrodes to produce nonthermal plasma and as efficient catalysts for ammonia synthesis under atmospheric pressure. Several metal wools were tested, the order of the activity at the initial experiment was Au > Pt > Pd > Ag > Cu > Fe > Mo > Ni > W > Ti > Al, however, do to the metal migration from the electrode to the inner wall of a silica reactor or increases in surface areas of metal catalysts, the catalytic activity of Pt, Pd, Ag, Cu and Ni wools increased as the experiments were repeated, while Au, Fe, Mo, Ti, W and Al shown almost a constant activity (Figure 7).

Herrera et al. [35] studied the effect of metal particles supported on Al_2_O_3_ on the macroscopic DBD plasma properties in an atmospheric pressure reactor. The study shows that, although the rates of ammonia synthesis over Fe/Al_2_O_3_, Ni/Al_2_O_3_ and Co/Al_2_O_3_ were different, the macroscopic properties of the DBD were statistically indistinguishable. These results suggested that the catalysts are not able to modify the macroscopic properties of the plasma, rather the plasma environment and the species generated by the plasma could modify the chemistry on the surface of the catalysts.

### 2.6. Reactor Configurations

Two interesting studies were also published on multireactor configuration, with the aim of improving the efficiency of the process. Akay [36] studied a catalytic multireaction–zone reactor (M–RZR) system, divided in two main zones (RZ-1 and RZ-2), for ammonia synthesis. The first zone (RZ-1) was used for the catalytic nonthermal plasma ammonia synthesis, which was immediately sequestrated by a highly porous polymeric solid acid absorbent in the neutralization reaction zone (RZ-2). The catalyst used were silica supported nickel or cobalt (with molar ratio M/Si = 1/4; where M = Ni or Co). The results shown that the ammonia conversion increased by a factor of 4 compared with no sequestration and the specific energy input (SEI) was reduced to 13.2 MJ mol^−1^ NH_3_, which was nearly 2−3 times smaller than those reported recently.

**Figure 8:**
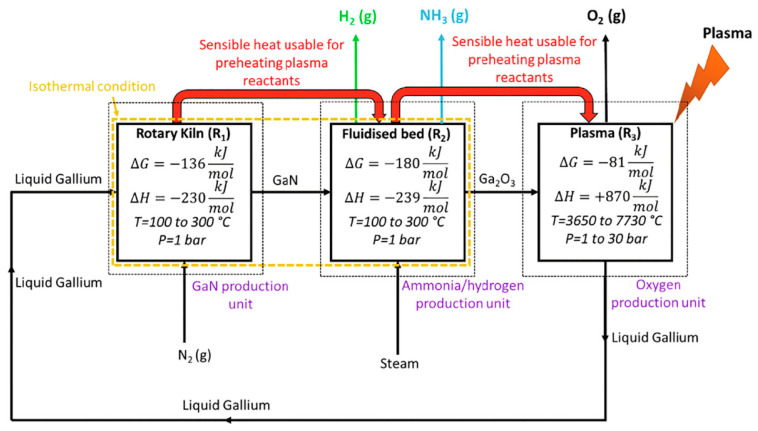
Schematic diagram of the process for the coproduction of ammonia and hydrogen using a plasma-assisted process [37]. Reproduced with permission from M.M. Sarafraz, N.N. Tran, N. Pourali, E.V. Rebrov, V. Hessel, Energy Conversion and Management; published by Elsevier, 2020.

Sarafraz et al. [37] carried out a thermochemical equilibrium analysis to assess the feasibility and the thermodynamic potential of a process for the coproduction of hydrogen and ammonia, by using liquid metal and a plasma reactor. The process takes advantage of a chemical looping system, liquid metal gallium drives the nitrogen fixation reaction, by using three reactors: R1 to produce gallium nitride from gallium and nitrogen, R2 to produce ammonia and hydrogen from gallium nitride and plasma reactor R3 to convert gallium oxide to pure gallium (Figure 8). The results shown that the proposed reactions are spontaneous, because the Gibbs free energy of the reactions in all reactors was negative, moreover the first two reactions are exothermic with ΔH = −230 kJ mol^−1^ (R1) and ΔH = −239 kJ mol^−1^ (R2), with an equilibrium conversion of 100%. The gallium oxide dissociation is endothermic reaction of with ΔH = +870 kJ mol^−1^ (plasma reactor R3), thus it requires energy, which can be obtained from R1 and R2. Thermodynamic analysis demonstrated that the reactor R2 can be used for the coproduction of hydrogen and ammonia, when the temperature and feed ratio of the reactor (φ) were in range 100 °C < T < 400 °C and 0.1 < φ < 1.0, at atmospheric pressure.

### 2.7. Urea Decomposition

Fan et al. [38] studied the effect of carrier gas composition (N_2_/air) on the ammonia synthesis from plasma–catalytic decomposition of urea, using an Al_2_O_3_-packed DBD reactor. The results shown that the presence of oxygen in the carrier gas accelerated the urea decomposition but decreased the ammonia selectivity. However, the use of air carrier gas reduced the energy consumption and increased the energy efficiency, if compared with pure N_2_. The carrier gas composition had a little influence on the decomposition pathway, when the carrier gas was nitrogen, small amount of N_2_O were produced, when the carrier gas was air, N_2_O and NO_2_ were produced in the gas phase and NH_4_NO_3_ was deposited as solid. At different power, the plasma-phase reactions shown a good correlation with the SEI (specific energy input), while, when the catalyst was present, keeping the SEI constant, higher ammonia production rates were obtained for higher input power, demonstrating the synergy between the plasma and the catalyst.

#### Conclusions

As already stressed in this short review on the recent advances on plasma-assisted ammonia synthesis, in principle ammonia production via NTP technology does not need a catalyst; however, the major issue with this process is the energy consumption. To overcome this limitation and make the process more efficient, a series of catalytic systems were developed, and innovative reactor configurations were proposed. Among the catalytic systems, for sure the best performance has been reported with the Ru-based catalysts, moreover the use of promoters such as magnesium oxide is able to significantly enhance the efficiency. On the other hand, multireactors can improve the efficiency, by exploiting the autothermal configuration or subtracting the produced ammonia, as soon it has been synthesized. A simple comparison between conventional processes and the plasma assisted process is risky, since while in the first case the target is widespread production, in the second case the target is distributed production. In any case, the plasma assisted process is far from being optimized, and further studies are needed to optimize the process; few catalytic systems have been investigated; therefore, this technology does not seem to be mature.

**Table 2:** Efficiency of selected catalysts for ammonia production via NTP technology.

Selected Catalyst	Reaction Conditions	Efficiency (g_NH3_ kWh^−1^)	Reference
Ru/Si–MCM-41	N_2_:H_2_ = 1:1; 5000 V	1.7	[23]
Ru/MCM-41	N_2_:H_2_ = 3:1; 6000 V	2.2	[24]
Ru(2)–Mg(5)/γ-Al_2_O_3_	N_2_:H_2_ = 4:1; T = 250 °C	25.5	[25]
Ni–MOF	N_2_:H_2_ = 1:4; T = 82.3 °C	0.23	[26]
PZT	N_2_:H_2_ = 3:1; 5000 V	0.65	[29]
DLC-coated Al_2_O_3_	N_2_:H_2_ = 3:1; 17,500 V	0.9	[30]
Zeolite 5A	N_2_:H_2_ = 1:1	15.5	[31]
Ni/Al_2_O_3_	N_2_:H_2_ = 1:2; 24,000 V	0.45	[32]

## 3. Catalytic SO_2_ Removal via NTP Technology

Various technologies have been developed for SO_2_ capture, including carbon-based material adsorption techniques, thermal catalytic conversion, wet flue gas desulfurization, wet scrubbing methods and plasma-based systems [39,40]. Among them, non-thermal plasma (NTP) technology is attracting increasing attention in the field of pollutants removal, due to the high oxidation rate, the fast and efficient takedown, its operability at atmospheric temperature and pressure, small flow area, absence of chemical additives and low operational as well as investment costs [41,42]. In order to reduce the SO_2_ emissions in industrial flue gases, NTP technology has also been investigated in combination with catalysts/photocatalysts systems as well as precipitators [43]. SO_2_ oxidation is carried out at high temperatures in the presence of Pt catalysts; however, the interaction of the gas phase with radicals produced via NTP technology in the presence of cheaper catalysts allows reducing the process temperatures [44]. NTP can be either generated by electron beam irradiation or electric discharge techniques (dielectric barrier, pulse and microwave) [45,46,47]. In this section, the application of some electron discharge techniques combined with catalysts and photocatalysts for SO_2_ removal will be discussed.

Cui et al. [41] described the performances of a dielectric barrier discharge (DBD) reactor, which is a kind nonthermal plasma reactor, for SO_2_ removal. In such reactor, electrical discharge is generated when a high voltage alternating current is imposed to the two electrodes, which are separated by one or more insulation dielectrics. This configuration allows a reduced energy consumption, a stable discharge and a more uniform plasma distribution compared with other NTP reactors. The DBD reactor was made up of four coaxial cylinders: a central stainless-steel rod, a stainless mesh tube, a corundum tube and a quartz tube (these latter two tubes acted as media). A wet electrostatic precipitator (WESP) was also added downstream the DBD reactor: the DBD process generates ·O, ·OH and O_3_ radicals, promoting the conversion of SO_2_ to H_2_SO_4_ or SO_3_ while water vapor favors the reaction of the oxidation products to form the corresponding acid-mists, also adsorbing them. When the only DBD reactor operates without oxygen feeding, SO_2_ conversion is extremely low (5%–8%), independently form the peak–peak voltage. Conversely, when oxygen is introduced, a linear increase of SO_2_ conversion with O_2_ concentration as well as peak–peak voltage growth (in the interval 3%–10% and 20–40 kV, respectively) was observed. However, SO_2_ removal efficiency (i.e., SO_2_ conversion) was lower than 50% for all the investigated rates, due to the quite low reaction rates between SO_2_ and the radicals ·O as well as O_3_. In the combined process, the generation of ·OH radicals and the higher concentration of ·O and O_2_ radicals increased the SO_2_ removal efficiency, which exceeded 95% at 38 kV under a 10%-O_2_/7%-H_2_O stream. In fact, it was reported that the reaction rate coefficient of SO_2_ with ·O and O_3_ radicals (3.52 × 10^−14^, 1.89 × 10^−22^ cm^3^ molecule^−1^ s^−1^, respectively) is much lower compared to the values recorded in the case of ·OH radicals (1.3 × 10^−12^ cm^3^ molecule^−1^ s^−1^) [43].

The same authors investigated the possibility of oxidizing and separating SO_2_ via a catalytic oxidation process [48]. In this regard, manganese oxide has been widely adopted, due to its redox capacity and strong adaptability; moreover, copper addition to MnO allows reducing the operating temperatures for oxidation. The calcination of the MnCu/TiO_2_ catalysts, prepared at an atomic ratio Mn/Cu of 1:1, was directly performed in the DBD reactor: compared to conventional calcination, reduced time is required to remove the templating agent and the precursor at a lower temperature; moreover, the reaction conditions favor the formation of surface defects and active sites, thus improving the catalyst reactivity. The results of XRD measurements revealed that the sample prepared by DBD was characterized by smaller crystallites and enhanced active phases dispersion compared to the MnCu/TiO_2_ catalyst prepared via the conventional calcination. For catalysts preparation, the discharge treatment was carried out for 90 min under a 10% O_2_ in N_2_ stream with a discharge frequency, effective voltage and discharge power of 8 kHz, 10 kV and 200 W, respectively. SO_2_ removal measurements were performed in the presence of NO. For the system combining the DBD reactor and the MnCu/TiO_2_ catalyst, the efficiency of SO_2_ removal decreased from 100%, recorded feeding 1000 mg m^−3^ of SO_2_ and 400 mg m^−3^ of NO, to 77%, measured under a stream containing 2000 mg m^−3^ of SO_2_ and 400 mg m^−3^ of NO. Moreover, despite SO_2_ is known to have a negative effect on the performance of Cu and Mn-based catalysts, a quite stable behavior for 120 min was recorded under a stream of 6% O_2_, 300 mg·m^−3^ NO and 1000 or 2000 mg·m^−3^ of SO_2_ at a specific energy density (SED, defined as the ratio between the discharge power in W and the gas flow rate in L s^−1^) of 275 J L^−1^; however, the increase of the total gas-flow rate from 4 to 8 L min^−1^, reduced the removal efficiency from 100% to 60% with an SO_2_ concentration of 1000 mg m^−3^. The performance of the DBD reactor–catalyst–WESP system was also investigated at different SED values: in this configuration, the SO_2_ removal efficiency increased with the SED growth and was enhanced with respect to that observed without the WESP system. Moreover, the SED required to reach a complete SO_2_ removal efficiency was reduced compared to the values needed in the same configuration without water vapor. The reaction involved in SO_2_ removal in the presence of water are described in Equations (2)–(6).

(2) $${\mathrm{SO}}_{2}+O◾\to {\mathrm{SO}}_{3}$$

(3) $${\mathrm{SO}}_{2}+O_{3}\to {\mathrm{SO}}_{3}+O_{2}$$

(4) $${\mathrm{SO}}_{2}+\mathrm{OH}◾\to {\mathrm{HOSO}}_{2}$$

(5) $${\mathrm{HOSO}}_{2}+O_{2}\to {\mathrm{SO}}_{3}+{\mathrm{HO}}_{2}$$

(6) $${\mathrm{SO}}_{3}+H_{2}O\to H_{2}{\mathrm{SO}}_{4}$$

They [49] also found that, in the presence of NO, SO_2_ and NO compete with each other in the removal mechanism; thus, SO_2_ removal efficiency in the presence of the MnCu/TiO_2_ catalyst can be enhanced by reducing the initial concentration of nitrogen monoxide. In fact, the radicals involved in the removal (·O and O_3_) are the same and the reaction rates of SO_2_ with such radicals (3.52 × 10^−14^, 1.89 × 10^−22^ cm^3^ molecule^−1^ s^−1^, respectively) are lessened compared to the values reported for NO (3.0 × 10^−11^ cm^3^ molecule^−1^ s^−1^ and 1.8 × 10^−14^ cm^3^ molecule^−1^ s^−1^, respectively) Thus, for a SED of 240 J L^−1^, SO_2_ removal efficiency grew from 70% to 90% by lowering NO concentration from 400 to 200 mg m^−3^.

Thus, coupling NTP technology with proper catalytic formulation is a suitable route to reduce, on one hand, the typical high temperature (400–900 °C) required for the thermal catalytic conversion of SO_2_, which promote surface sulfate formation and catalyst deactivation [50] and to increase, on the other hand, the selectivity of nonthermal plasma process. In this regard, various hybrid systems containing catalysts as well as photocatalysts have been investigated.

**Figure 9:**
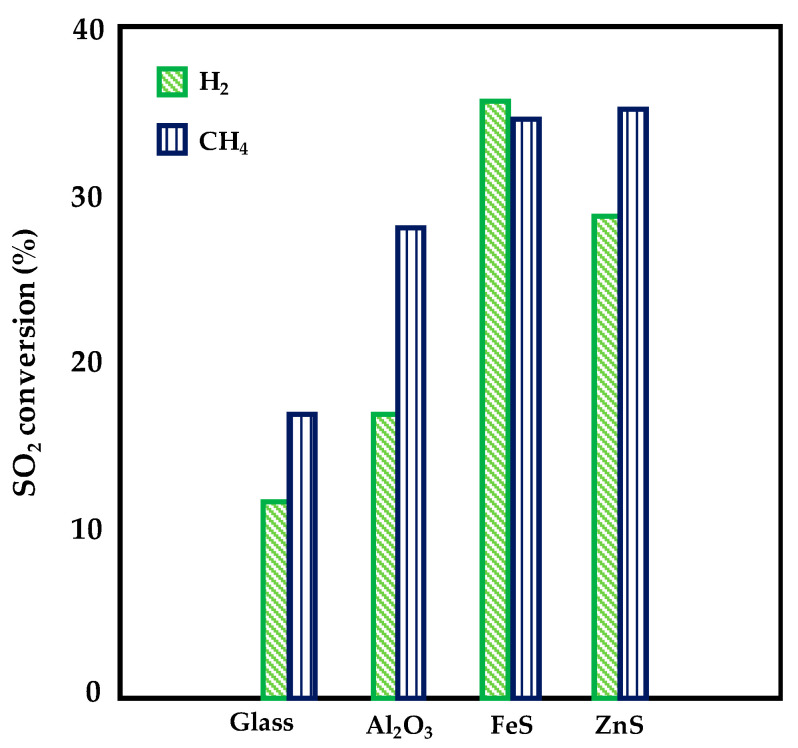
SO_2_ conversion with various packings [51]; 1% SO_2_, 4% H_2_ or CH_4_, N_2_ balance, flow rate = 100 mL min^−1^ at 150 °C, 1 atm and 10-W DBD plasma. Adapted with permission from Mohammad S. AlQahtani, Sean D. Knecht, Xiaoxing Wang, et al., ACS Catalysis; published by American Chemical Society, 2020.

AlQahtani et al. [51] investigated the direct conversion of SO_2_ to elemental sulfur (easier to be handled and free of the corrosion issue typical of sulfuric acid) in a DBD reactor loaded with Fe and Zn sulfide catalysts (supported on alumina). The study was performed at 150 °C and atmospheric pressure; hydrogen and methane were used as reductant agents. Despite catalysts are inactive at such low temperatures, which normally render SO_2_ conversion not kinetically feasible, reaction however occurred due to the plasma effect. The reactor was made up of a pair of glass tubes, inner quartz tube and outer borosilicate tube. The catalysts were prepared by wet impregnation and the iron as well as zinc loading were fixed to 10 wt%; the sulfidation of the catalysts was carried out under plasma at 10 W and 150 °C for 1 h. Both the samples displayed a specific surface area of 140 m^2^·g^−1^ with a good active species dispersion. The performances of the DBD reactor in the presence of FeS/Al_2_O_3_ and ZnS/Al_2_O_3_ catalysts were compared with the results recorded with a glass packing as well as with bare alumina. Under a 100 mL min^−1^ of a nitrogen stream containing 1% SO_2_, 4% H_2_ or CH_4_ and 10 W DBD plasma, SO_2_ reduction in the presence of H_2_ over the FeS/Al_2_O_3_ catalyst was promoted by 200% compared to the glass packing; both the catalysts increased SO_2_ conversion by a factor of 2 during CH_4_ co-feeding with respect to the case of glass packing. Compared to the results obtained with the alumina support, H_2_ conversion was promoted by 81% over the FeS/Al_2_O_3_ catalyst and 69% over the ZnS/Al_2_O_3_, with an increase in SO_2_ conversion of 109% and 69%, respectively (Figure 9); the eventually formed H_2_S is converted to elemental S in the presence of the remaining SO_2_ via the Claus reaction.

The reduced performances of the Zn-based catalysts were ascribed to the value of bond dissociation energy (225 kJ mol^−1^, compared to 335 kJ mol^−1^ of the Ir-based catalyst), which lead to a harder regeneration of the sulfur vacancies of the catalyst. The reaction occurring in the hybrid system are reported in Equations (7)–(11).

(7) $${\mathrm{SO}}_{2}{}_{\left(g\right)}+2H_{2}{}_{\left(g\right)}\to S_{\left(l\right)}+2{\;H}_{2}O_{\left(g\right)}$$

(8) $$2{\;\mathrm{SO}}_{2}{}_{\left(g\right)}+{\mathrm{CH}}_{4}{}_{\left(g\right)}\to 2{\;S}_{\left(l\right)}+{\mathrm{CO}}_{2}{}_{\left(g\right)}+2{\;H}_{2}O_{\left(g\right)}$$

(9) $${\mathrm{SO}}_{2}{}_{\left(g\right)}+{\mathrm{CH}}_{4}{}_{\left(g\right)}↔H_{2}S_{\left(g\right)}+{\mathrm{CO}}_{\left(g\right)}+H_{2}O_{\left(g\right)}$$

(10) $${\mathrm{SO}}_{2}{}_{\left(g\right)}+3{\;H}_{2}{}_{\left(g\right)}↔H_{2}S_{\left(g\right)}+2{\;H}_{2}O_{\left(g\right)}$$

(11) $${\mathrm{SO}}_{2}{}_{\left(g\right)}+2{\;H}_{2}S_{\left(g\right)}↔3{\;S}_{\left(l\right)}+2{\;H}_{2}O_{\left(g\right)}$$

In the combined NTP–TiO_2_ photocatalysts systems, the nonthermal plasma can be a source of the ultraviolet light for the activation of titania: photons irradiation allows generating electrons and holes, which act as reductants in SO_2_ removal. For the DBD-TiO_2_ photocatalyst technology, the dielectric glass beads are coated by TiO_2_ and the uniform deposition of such coating is crucial to reach high efficiency removal of sulfur dioxide. In this regard, different methodologies of titania deposition have been proposed.

A TiO_2_ photocatalyst was deposited on the glass beads via the dip-coating method and SO_2_ removal was investigated in a two-zones reactor: in the first zone (packed with the coated glass beads), SO_2_ conversion to SO_3_ and its further reaction with ·OH radicals for sulfuric acid generation occurred while in the second zone ammonium sulfate particles were formed by the neutralization reaction between H_2_SO_4_ and NH_3_ and grew inside the reactor [52]. Such particles can be easily separated by a particle collector. In such system, SO_2_ removal efficiency was investigated under an initial water concentration of 400 ppm, fed with an air stream and SO_2_; the ammonia concentration was 1 half of the SO_2_ fed. The initial SO_2_ concentration, pulse frequency, applied voltage, input power and total gas flow rate were varied in the ranges 200–600 ppm, 100–900 Hz, 3–13 kV, 3–50 W and 2.5–10 L min^−1^ (corresponding to residence times of 1–0.32 s), respectively. A growth in the applied voltage resulted in a faster oxidation rate and a quicker SO_2_ removal. When SO_2_ concentration is higher, the energy required for its removal increases and, at a fixed applied voltage, the efficiency of the removal is lessened. Under the selected experimental conditions, the specific energy consumption (defined as the ratio between the power and the converted flowrate) for sulfur dioxide removal was in the interval 190–530 eV molecule^−1^. An increase in the pulse frequency also resulted in an enhanced SO_2_ removal efficiency. For a residence time of 1 s, at 8.5 kV and 900 Hz, the SO_2_ removal efficiency increased from 30 (recorded at 0.32 and 0.5 s) to 80%. Concerning the second zone of the reactor, the dimension of (NH_4_)_2_SO_4_ particles (which was lower than 1 µm under the selected operative conditions), analyzed via TEM method, grew by increasing the reactor length as well as the initial SO_2_ concentration and reducing the total gas flow rate.

Nasonova et al. [53] coated TiO_2_ thin films on the glass beads (3-mm-diameter, acting as dielectric material) by the rotating cylindrical PCVD (plasma chemical vapor deposition) reactor and investigated SO_2_ removal in a NTP–DBD reactor packed with the TiO_2_-coated glass beads. A cylinder–wire type pellet packed-bed reactor was used, with a copper road wire located at the center of the glass tube (powered electrode); the outside wall of the reactor was wrapped with stainless steel mesh (ground electrode). Concerning the TiO_2_ deposition, TEM analysis revealed that the coating procedure allowed the absence of cracks in the layer, having a thickness of 150 nm. Furthermore, in this case, the experiments were performed in the presence of NO and the following operative conditions were fixed: 1 atm, 25 °C, 750 ppm of NO, 420 ppm of SO_2_, 21% O_2_ and N_2_ for balance, pulse frequency of 300–900 Hz, residence time of gas stream of 0.5–2 s; the applied peak voltage varied from 3 to 13 kV and the tests were performed at 1 atm and 25 °C. High frequencies and increased applied peak voltage enhanced the number of micro-discharges between the packed dielectric material as well as the electron energy for radical generation and, as a consequence, the concentration of reactive radicals, thus resulting in a growth in the removal efficiency of the sulfur dioxide. The plasma reactions for SO_2_ removal are also favored at higher gas residence times. The performance of the catalyst prepared by the PCVD method was compared with that of a sample obtained by dip-coating the glass beads with a commercial titania catalyst: the thin film deposited via the PCV technique was more uniform and characterized by a large surface area. Thus, at 9 kV and 900 Hz, the SO_2_ removal efficiency increased from 45% to 67%.

Similarly, Pham & Kim [54] deposited thin TiO_2_ films on the glass beads by a rotating cylindrical plasma chemical vapor deposition reactor and investigated the effect of the thickness of such films on SO_2_ removal efficiency. They found that it was possible to precisely control the film thickness as it is proportional to the deposition time (different films were prepared at fixed deposition times: 3, 10, 30, 45, 60, 70, 80 min): by changing the deposition time from 3 to 80 min, the thickness of the films grew from 35 to 820 nm, as attested by the SEM analysis of the cross-section of the films. The influence of film thickness on the SO_2_ removal efficiency was studied under an air stream at an initial concentration of SO_2_ of 260 ppm, a pulse frequency of 900 Hz, a residence time of 1 s and an applied peak voltage in the interval 5–15 kV. The reactor configuration was the same described above for the works of Nasonova et al. [53]. SO_2_ removal efficiency displayed an increasing trend with the applied peak voltage, whatever the selected film thickness; however, such efficiency grew with TiO_2_ thickness until the value of 600 nm and then, a decrease in SO_2_ conversion with a further thickness rise was recorded. In fact, for too thin films, part of the UV light penetrates the glass beads, without being utilized to generate the charge carriers transported towards the TiO_2_ surface for its activation. Similarly, for too thick films, the TiO_2_ light adsorption reaches a saturation level: the time for the migration of the charge-carriers is too high and their recombination becomes more competitive than their transportation towards the surface. Thus, an optimal value of 600 nm for the TiO_2_ thickness was identified, which allowed reaching an almost complete SO_2_ conversion at 11 kV. At the same applied voltage, the SO_2_ removal efficiency was lower than 20% for the films of 32 and 90 nm and equal to 95% for the film having a thickness of 820 nm.

TiO_2_ films were also deposited on zeolite particles via the PCVD technique described above; the performance of zeolite (acting as catalyst) and TiO_2_-coated zeolite (acting as photocatalyst) was investigated for SO_2_ removal in a dielectric barrier discharge hybrid process [55]; zeolite particles were used as dielectric material and catalysts for the DBD. For the TiO_2_ coating of the zeolite particles, the following conditions were fixed: deposition time, mass flow rate of titanium tetra-isopropoxide, applied power, reactor pressure, and rotation speed of the reactor were 30 min, 3.45 mg min^−1^, 30 W, 1 atm and 30 rpm, respectively. The deposition process was properly studied in order to cover only partially the zeolite surface, thus preserving its high surface area and avoiding the blockage of its pores. SO_2_ removal efficiency was investigated in the presence of the catalyst and the photocatalyst with an initial SO_2_ concentration in the range 200–400 ppm; the applied peak voltage, the pulse frequency and the residence time were in the interval 7–15 kV, 300–900 kHz, 0.5–2 s, respectively. The configuration of the reactor employed in this work has been previously described [53]. Whatever the selected operative conditions, the SO_2_ removal efficiency for the TiO_2_-coated zeolites is higher compared to the values recorded in the presence of titania-free catalyst, due to the enhanced generation of the reactive radicals over the photocatalyst, which is able to exploit the UV light generated inside the NTP reactor. Such differences became higher at intermediate applied voltage (10–13 kV). One hundred percent SO_2_ conversion was recorded at 14 kV, 900 Hz, 1 s and 200 ppm of SO_2_ for the hybrid system containing the photocatalyst while a value of only 77% was measured when the zeolite catalyst was used. At 400 ppm of SO_2_ and 900 Hz, the highest efficiency removal was recorded at 2 s over both the catalytic systems above 12 kV. However, at 13 kV, the TiO_2_ coating allowed increasing sulfur dioxide conversion from 38% to 58%. Finally, the effect of pulse frequency variation on SO_2_ conversion was little evident for every series and the profiles recorded over the coated sample were still higher than those measured over the uncoated zeolite particles.

Wang et al. [56] investigated SO_2_ removal in a hybrid system using a DBD reactor and γ-Al_2_O_3_ (specific surface area of 280 m^2^·g^−1^) as adsorbent. The reactor, packed with alumina pellets into the discharge area, consisted of two coaxial quartz tubes, with an aluminum foil paper and a stainless-steel rod acting as the ground electrode and high voltage electrode, respectively. The measurements were performed with the reactor operating at a discharge power of 5.5–7.5 W under a 10% O_2_ in nitrogen stream (600 mL min^−1^) containing 460 ppm of SO_2_. The tests were carried out both on alumina after treating it with NTP and by using NTP as the source of radiation. In the first case, the pretreatment had a negligible impact on the SO_2_ adsorption capacity of alumina. In fact, sulfur oxide itself can be strongly adsorbed on γ-Al_2_O_3_. Thus, when the adsorption is studied in the presence of 440 ppm of NO and 14 ppm of NO_2_, in the initial stages, both NO_x_ and SO_2_ can adsorb on the free alumina sites; however, with the reduction of the number of free adsorption sites, a competition takes place ad SO_2_ (having higher affinity with the alumina) displaces the NO_x_ molecules. For the hybrid system using the NTP as source of radiation, the oxidation efficiency for SO_2_ shown a maximum (corresponding to a 20% of conversion) with discharge power variation in the interval 5.5–7.5 W. Conversely, the oxidation efficiency of NO is enhanced (50%), due to the higher reaction rates of the nitrogen monoxide [49]. Anyway, the interactions between the radicals formed upon NTP irradiation and alumina assured the generation of adsorbed oxygen atoms on the surface, which enhanced SO_2_ adsorption and sulfate formation.

Besides the DBD reactor, another NTP technology used for pollutants removal involves the pulsed streamer corona plasma, which are characterized by high safety, low cost and high discharge efficiency [57].

Kim et al. [44] studied the oxidation of SO_2_ to SO_3_ in a nonthermal plasma hybrid reactor based on the pulsed-corona plasma (PCP) and operating with TiO_2_ pellets (3 mm) as catalyst in the presence of H_2_O or H_2_O_2_. The reactor was made up of a cylindrical quartz tube; the high-voltage power supply was equipped with the positive electrode, placed inside the quartz reactor and the negative electrode, placed on the outside of the quartz reactor. The gas-phase reaction induced by plasma occurred in a precise zone of the reactor, consisting of a metal tube electrode, located in the center of the quartz reactor and a copper thin-film ground electrode, wired around the quartz reactor; the first one electrode was also used to supply the additives (water and hydrogen peroxide).

**Figure 10:**
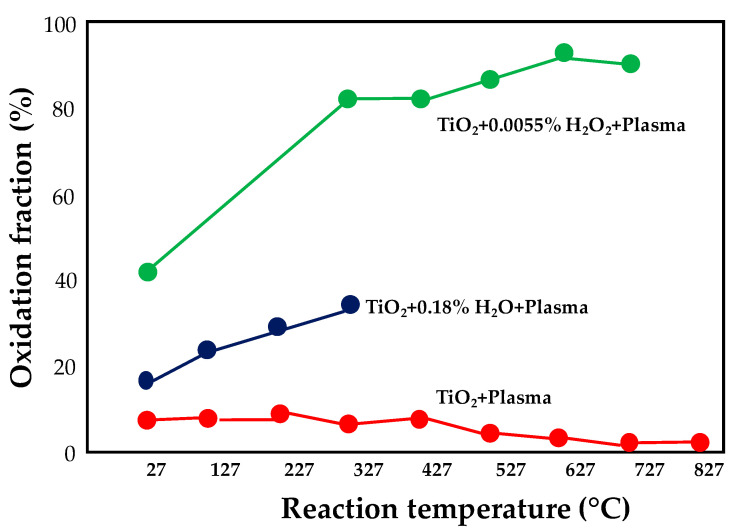
SO_2_ oxidation fraction for a pulsed streamer corona plasma in the presence of TiO_2_ and various additives [44]; flowrate of 1 L min^−1^, 906 ppm (N_2_ basis), O_2_: 2.1%; H_2_O: 0.18%; H_2_O_2_: 0.055%; plasma applied of 12.5 kV, 1 atm, 25 °C. Adapted with permission from Heejoon Kim, Akira Mizuno, Yuhei Sakaguchi, et al., Energy & Fuels; published by American Chemical Society, 2002.

A second zone of the reactor was packed with TiO_2_ and was devoted to the surface reactions. SO_2_ oxidation measurements were performed between 25 and 800 °C under a stream (1 L·min^−1^) having the following composition: SO_2_: 906 ppm (N_2_ basis); O_2_: 2.1%; H_2_O: 0.18%; H_2_O_2_: 0.055%; plasma was applied at 12.5 kV. H_2_O as well as H_2_O_2_ additives promote the formation of ·OH radicals, which play an important role in SO_2_ oxidation to SO_3_. However, due to its higher reactivity, hydrogen peroxide can be easily decomposed to produce OH in the gas phase as well as on the TiO_2_ surface. The variation of the SO_2_ oxidation fraction as a function of temperature in the hybrid system with and without additives is reported in Figure 10: water addition resulted in an enhancement of the oxidation of almost 10% while an improvement of 30%–35% was recorded in the presence of H_2_O_2_.

Table 3 summarizes the above results in terms of SO_2_ conversion recorded during NTP in the presence of various catalysts as well as photocatalysts.

## 4. Catalytic H_2_S Removal via NTP Technology

H_2_S, a common contaminant of natural gas also deriving from sewage treatments and solid waste disposal facilities [58], can be used as source of hydrogen and different processes are available for its conversion. The most widespread technology for H_2_S removal is the commercial Claus process which, however, leads to H_2_O formation (instead of H_2_) and is not capable to reach complete conversion. Thus, various alternative techniques, including wet scrubbing, adsorption, thermo catalytic, electrochemical and plasma-assisted methods have been investigated [59,60]. Among them, the nonthermal plasma technology offers a direct and clean approach and has been proposed as a suitable alternative for direct decomposition of H_2_S to H_2_ and S, especially due to the achievement of high electron energies within a short residence time and the rapidity of the reactions at ambient conditions [61,62]. Nonthermal methods including dielectric barrier, corona, rotating glow, microwave, gliding arc discharge and radio frequency discharge have been studied for H_2_S removal [63,64,65]. In the present section, the application of dielectric barrier discharge techniques combined with catalysts as well as photocatalysts for H_2_S removal will be discussed.

The performances of various DBD reactor–catalyst hybrid systems have been investigated for H_2_S decomposition and, in many cases, alumina was selected as catalytic support.

Zhao et al. [66] studied H_2_S conversion in a DBD reactor consisting of a quartz tube and two electrodes (high-voltage electrode was made of stainless steel while the grounding electrode was an aluminum foil wrapped around the quartz tube); the catalysts (alumina-supported semiconductors) can be placed into the gap between the quartz tube and the high-voltage electrode. Al_2_O_3_-supported CdS, ZnS and Zn_0.4_Cd_0.6_S were used; oxide precursor was loaded on the support and sulfiding process was performed at 400 °C for 180 min in a 10% H_2_S/Ar flow. The metals sulfide loading was varied between 1 wt% and 30 wt%. The reactor was maintained at 120 °C to allow the formation of sulfur liquid drops and prevent its deposition on catalyst surface. The discharge power was varied between 0 and 7 W, with a frequency of discharge of 10 kHz, while the H_2_S concentration in the feeding stream was 20% in argon. The results of catalysts characterization revealed that, by reducing the metals sulfide loading, it is possible to increase the surface area as well as the active phases dispersion. Thus, the 1% CdS/Al_2_O_3_ catalyst displayed a specific surface area of 263 m^2^ g^−1^ and an average size of crystallites of 4.3 nm, while values of 266 m^2^ g^−1^ and 3.3 nm were recorded for the Zn-based sample. According to the pathway reported in Equations (12)–(16), the H_2_S decomposition induced by NTP involves the formation of free radicals and ion radicals.

(12) $$H_{2}S↔\mathrm{HS}+H$$

(13) $$H+H_{2}S↔\mathrm{HS}+H_{2}$$

(14) $$\mathrm{HS}+\mathrm{HS}↔{\;H}_{2}S+S$$

(15) $$H+\mathrm{HS}↔{\;H}_{2}+S$$

(16) $$S+\mathrm{HS}↔{\;S}_{2}+H$$

For an H_2_S concentration of 20% and a SED of 5.93 kJ L^−1^, H_2_S conversion was 97.9%, 90.9% and 82.9% when CdS/Al_2_O_3_, ZnS/Al_2_O_3_, Al_2_O_3_ were filled in the gap, respectively. Conversely, for the empty gap, the conversion was 54%. The presence of a packing, in fact, increases the residence time of the plasma-generated active species and enhances the local field as well as the electric discharge. For the Cd and Zn semiconductors, the H_2_S conversion increased monotonically with the SED and the maximum conversion was recorded at 5.93 and 8.14 kJ L^−1^, respectively: the presence of the catalysts accelerated the rate-determining steps in the secondary reactions (Equations (13)–(16)). The photons generated by NTP and the strong electrical field excite the semiconductors and generate hole–electron pairs: the holes oxidize H_2_S or SH to form H^+^ while the electron reacts with H^+^ to generate H_2_. By increasing the metals loading under an H_2_S concentration of 20%, a growth in H_2_S decomposition rate was observed, with a maximum activity at 10% for both Cd and Zn-based catalysts. The worsening in the performances observed for higher loadings is ascribed to the reduced surface area and the increased metal particles. An enhancement in H_2_S conversion with respect to the values recorded over the Zn- and Cd-based samples was measured in the case of the Zn_0.4_Cd_0.6_S/Al_2_O_3_ solid solution, ascribable to the fact the solid solution possesses a favorable band gap and a moderate position of conduction band. Under a GHSV of 120 h^−1^ and for a dissociation energy costs of 6 eV/H_2_, the solid solution reached complete H_2_S conversion, while values of 90% and 88% were recorded for the Cd- and Zn-based semiconductors, respectively. The durability of the CdS/Al_2_O_3_ and ZnS/Al_2_O_3_ was also investigated at 6 and 8 kJ L^−1^ [67], respectively, finding a stable behavior for 100 h in both cases. The lower activity of Zn-based catalysts compared to the Cd containing samples was ascribed to its larger band gap and a lower population of h^+^/e^−^ pairs. CdS and ZnS semiconductors can also be excited by UV and visible light, acting as photocatalysts. The contribution of photocatalysis was investigated in a modified reactor, with an upper part for the generation of nonthermal plasma by DBD (the discharge gap was empty) and a lower part consisting of a fixed bed of catalyst, irradiated with a UV lamp; the two parts were separated by a thin layer of quartz wool. When the UV lamp was off, the H_2_S conversion profiles over the two samples as a function of the applied voltage (9–12 kV) were remarkably close, proving that H_2_S decomposition did not occur on the catalysts. Conversely, in the presence of the UV lamp, a significant improvement in H_2_S conversion was observed, which reached 50% over CdS and 45% over ZnS at 12 kV, due to the synergy between the electrical field and the light irradiation.

The same authors [68] investigated the effect of Zn/Cd molar ratio on the performance of ZnCdS/Al_2_O_3_ semiconductors for H_2_S decomposition in the DBD reactor described above. For the Zn_x_Cd_1−x_S/Al_2_O_3_, XRD analysis revealed that the average particle size of solid solution crystalline domain decreases from 9.4 to 8.1 nm with a growth in Zn content up to 0.6, which is ascribable to the presence of Zn in the CdS crystal. The dimension of the nanoparticles was confirmed by TEM analysis. The Zn/Cd molar ratio also affected the band gap of the solid solution, investigated by UV-visible spectroscopy analysis: Zn content monotonically increased the band gap size, which promotes the charge separation in h^+^/e^−^ pairs and avoid their recombination in the bulk semiconductor. Thus, a growth in Zn concentration from 0.2 to 0.6 increased the catalyst activity towards H_2_S decomposition; a subsequent increase up to 0.8 resulted in a worsening of the performances. These results demonstrate that, besides the band gap size, the physiochemical properties of the catalyst also affect its activity for H_2_S decomposition: small particle sizes assure a faster transportation of the electron from the bulk towards the surface and results in a higher number of effective reaction sites. Therefore, for an energy consumption of 6.12 eV/H_2_, H_2_S conversions were 100%, 97.9%, 92.8% and 84.9% for the Zn_0.6_Cd_0.4_S/Al_2_O_3_, Zn_0.4_Cd_0.6_S/Al_2_O_3_, Zn_0.2_Cd_0.8_S/Al_2_O_3_Zn_0.8_Cd_0.2_S/Al_2_O_3_ catalysts, respectively.

**Figure 11:**
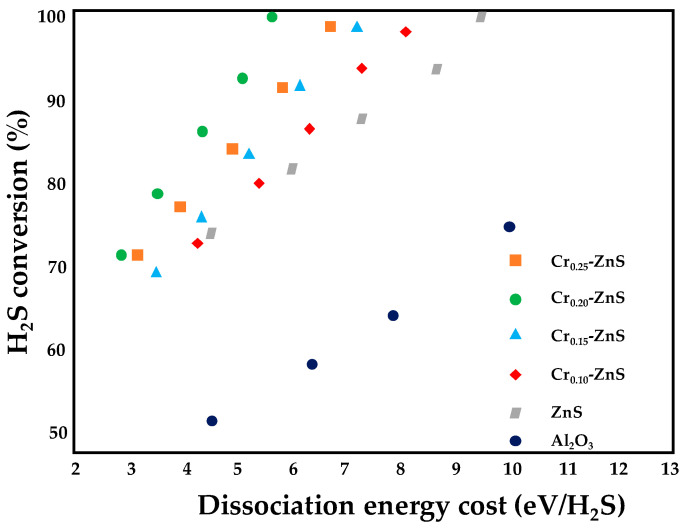
H_2_S conversion over Crx–ZnS/Al_2_O_3_ catalysts in a DBD reactor [69]; 10 kHz, 20 vol% H_2_S in Ar, GHSV = 120 h^−1^, 1 atm, 120 °C. Adapted with permission fromLu Zhao, Yao Wang, Anjie Wang, Xiang Li, Chunshan Song, Yongkang Hu, Catalysis Today; published by Elsevier, 2019.

The ZnS/Al_2_O_3_ catalyst was also doped by different metal ions, finding that the H_2_S conversion increased in the order Fe < Co < Ag < Ni < W < Cu < Mo < Ce < Mn < Cr [69]. Thus, Cr exhibited the highest activity and the influence of chromium doping on the performance of the ZnS semiconductors for H_2_S removal via NTP was studied; the reactor configuration was the same described above (the discharge frequency was fixed to 10 kH while the applied voltage changed from 0 to 15 kV) [66]. The catalysts were prepared by wet impregnation, followed by sulfuration; the ZnS loading was fixed to 10 wt% while the Cr/Zn ratio ranged from 0 to 0.25. The results of XRD analysis, confirmed by TEM measurements, revealed that a growth in the Cr/Zn molar ratio was accompanied by a slight decrease in the average particle sizes (from 9.3 at a ratio of 0.2 to 7.8 at a ratio of 0.25): an increase in the Cr content enhanced the lattice defects of ZnS, thus decreasing ZnS crystallinity. Moreover, higher Cr contents resulted in a decrease of band gap size, thus affecting the relative redox ability of the Cr-doped catalysts. The catalytic performance of the Cr_x_–ZnS/Al_2_O_3_ catalysts in the DBD reactor was investigated as a function of the dissociation energy cost and the results are shown in Figure 11. All the doped catalysts displayed improved activity compared to the Cr-free catalysts and the bare support; moreover, the H_2_S conversion increased with Cr loading up to 0.20. In fact, as discussed above for the Cd-doped catalysts, a decrease in the band gap assures a growth in the population of hole–electron pairs; moreover, small nanoparticles with poor crystallinity favor the fast electron transportation from bulk to surface, avoiding, at the same time, the hole–electron recombination. Thus, for an energy consumption of 5.57 eV/H_2_S, hydrogen sulfide conversion was 100%, 89.7%, 87.4% and 81.8% for x = 0.20, 0.25, 0.15 and 0.10, respectively.

The activity of the Cr_0.20_–ZnS/Al_2_O_3_ catalyst was also investigated as a function of the SED at a concentration of H_2_S ranging from 20% to 40%, finding that hydrogen sulfide conversion can be maximized at low concentrations. Under these conditions, in fact, a large part of the electrons collide with Ar balance gas, which plays a key role in decomposition reactions. Thus, for a SED of 4 kJ L^−1^ and a GHSV of 120 h^−1^, the H_2_S conversion passed from 55% at 40% of sulfur dioxide to 90% at a concentration of 20%. Moreover, the long term stability of the most active catalyst (Cr_0.20_–ZnS/Al_2_O_3_) was also investigated under a 20% H_2_S/Ar stream, a GHSV of 120 h^−1^ and a SED of 5.5 kJ L^−1^, observing no deactivation during 5 h of test.

Zhao et al. [70] studied the activity of MoS_2_/Al_2_O_3_ catalysts for H_2_S–CO_2_ conversion to syngas in the DBD reactor previously described [65]. The catalysts were prepared via wet impregnation with 1 wt%–15 wt% loading of the oxide precursor; thereafter, sulfuration occurred. Activity measurements were performed at 10 kHz, with an applied voltage between 0 and 20 kV under a feed flowrate of 35–70 mL min^−1^ and an H_2_S/CO_2_ molar ratio of 30/5, 25/10, 20/15, 15/20, 10/25, 5/30. In the presence of carbon dioxide, water and elemental sulfur were simultaneously generated (Equation (17)):

(17) $$2H_{2}S+{\mathrm{CO}}_{2}\to \;\mathrm{CO}+{\;H}_{2}+2S+H_{2}O$$

XRD as well as BET measurements revealed crystallite sizes of 4.4, 6.2, 8.6 and 10.5 nm and specific surface areas of 293, 265, 245 and 224 m^2^ g^−1^ for MoS_2_ loadings were 1%, 5%, 10% and 15%, respectively. Thus, higher MoS_2_ contents increased the particle sizes and decreased the areas. H_2_S as well as CO_2_ conversion increased over the 5-wt% MoS_2_/Al_2_O_3_ catalyst with the SED: under a feed of 35 mL min^−1^ and an H_2_S/CO_2_ ratio of 20:15, complete hydrogen sulfide conversion was recorded for a SED of 110 and 130 kJ L^−1^, with corresponding CO_2_ conversions of 56% and 63%, respectively. At a SED of 95 kJ L^−1^, only a slight reduction in H_2_S conversion (from 100% to 98.1%) was observed upon the increase of feed flow rate from 35 to 70 mL min^−1^. Thus, the dependence of the activity on the energy input, associated with the population of reactive species, is much more evident compared to the feeding rate. Concerning the effect of feed gas composition, under a flow rate of 35 mL·min^−1^, H_2_ conversion higher than 95% were recorded for a SED of 30–120 kJ L^−1^ under a H_2_S/CO_2_ ratio of 5:30. The activity of the MoS_2_-based catalysts, under a flow rate of 35 mL min^−1^ and a H_2_S/CO_2_ ratio of 20:15, increased with the MoS_2_ loading up to 5 wt%; for higher loadings, H_2_S conversion decreased due to lower surface areas and larger particle sizes. During 50 h of stability tests at 85 kJ L^−1^, 35 mL min^−1^ and H_2_S/CO_2_ ratio of 20:15, the most active catalyst also displayed a high sulfur tolerance with a quite stable hydrogen sulfide conversion (98%–100%) and more than 60% CO_2_ conversion.

The activity of MoO_x_/Al_2_O_3_ catalysts (with a MoO_x_ loading of 3 wt%, 5 wt% and 7 wt%) for H_2_S conversion in a DBD reactor was also investigated [71]. The reactor was made up of a cylindrical quartz tube with silver paste painted on its outside (acting as outer electrode) and a stainless-steel road (acting as inner electrode). The frequency was of 50 Hz and the applied voltage was in the interval 12–22 kV; the H_2_S inlet concentration ranged from 5% to 25% in Ar (total flow rate of 150 mL min^−1^). Whatever the initial H_2_S concentration, its conversion linearly increased with the input power; however, a growth in the inlet concentration caused a reduced activity: for example, at 1 W, the conversion decreased from 48% (at 5% of H_2_S) to 8% (at 25% of H_2_S). For an initial H_2_S concentration of 5%, the highest activity was recorded over the 5-wt% loaded catalyst. This result can be explained considering that the catalyst with a loading of 3% is fast poisoned by sulfur while for a 7% content of MoO_x_ the high oxide loading may change the discharge behavior. Thus, at 1 W, the 5MoO_x_/Al_2_O_3_ catalyst reached a conversion of 52% while values of 48% and 45% were recorded over the 3-wt% and 7-wt% samples, respectively.

Ning et al. [72] studied H_2_S oxidation over a catalyst made of iron oxide supported on walnut-shell activated carbon (Fe/WSAC) in a DBD reactor. The coaxial cylinder-type reactor was composed by a dielectric barrier (made of quartz) located between a stainless-steel tube (high voltage electrode) and a grounded electrode wrapped on the outer wall. The diameter of the high voltage electrode can be changed, thus controlling the size of the discharge gap. Measurements were performed with a pulse frequency of 7.8 kHz and a discharge voltage of 5.6–8 kV; a thermostatic water bath allowed the system to maintain a temperature of 60 °C. A N_2_ gas mixture with 500 ppm of H_2_S was fed with a total flow-rate of 60 mL·min^−1^ and a GHSV = 60,000 h^−1^. The catalyst was prepared by loading 5 wt% of iron oxide on the activated carbon (by coprecipitation). KOH was also added at a ratio KOH/WSAC of 13%: the presence of alkaline metals on the activated carbon surface, in fact, promotes H_2_S removal. Thereafter, the catalyst was subjected to a NTP treatment for different times (5, 7.5, 10, 12.5 and 15 min) at different output voltages (5.6, 6.2, 6.8, 7.4 and 8.0 kV) under an NH_3_ stream of 60 mL min^−1^. The effect of output voltage was investigated at a fixed time of 10 min and the results revealed that all the treated samples were able to maintain complete H_2_S conversion for higher time compared to the unmodified catalyst; however, the best performances were recorded for a voltage of 6.8 kV. In fact, the NTP treatment modifies the population of oxygen functional groups on the catalyst surface and such effect increases the oxidation capability up to 6.8 kV. For higher voltages, the too high number of oxidizing species can destroy the surface of activated carbon, thus worsening the catalytic performances. At a fixed voltage of 6.8 kV, the effect of the time treatment was also studied, finding the optimal activity upon 10 min of treatment. For longer times, in fact, the collision among the enhanced ions can cause etching on the activated carbon and pore collapsing. The BET measurements, in fact, revealed that the catalyst treated for 10 min at 6.8 kV displayed the largest pore volume (0.2954 cm^3^ g^−1^) while high voltages as well as long treatment times caused the surface destruction and the clogging of the pores. The results of XPS analysis also revealed that the NTP treatment affects the functional groups on the catalyst surface, favoring the transformation of the chemisorbed oxygen into lattice oxygen, which reacts with H_2_S. Moreover, the amino groups, introduced by NH_3_ during the treatment, can react with H_2_S forming NH_2_-HS; then, the lattice oxygen can oxidize the sulfur bond, promoting the formation of SO_3_^2−^ and SO_4_^2−^.

The same authors [73] investigated the influence of gas gap (3.5, 4.5, 5.5, 6.5, 7.5 mm) at a fixed dielectric thickness of 1.5 mm and dielectric thickness (1, 1.5 and 2 mm) at a fixed gas gap of 5.5 mm during the NH_3_ treatment on the performance of the Fe/WSAC for H_2_S removal in the DBD reactor described above. For these catalysts, the treatment was performed at 6.8 kV for 10 min. For a dielectric thickness of 5.5 mm, a complete conversion was maintained for 210 min while the untreated sample displayed 100% conversion only for 30 min. Increasing the gas gap, in fact, assures a more powerful discharge intensity, thus enhancing the strength of the interaction between the reactive species. However, a further increase of the gas gap above 5.5 mm may cause damages on the catalyst surface. The dielectric role in the DBD reactor is to avoid arc or spark generation in the discharge gap and dielectric thickness affects the strength of the discharge. Thus, H_2_S conversion grew with the thickness increase up to 1.5 mm while a decreasing trend was caused by a further increase in the thickness. In fact, the thickness of 1 mm is too low and thinner dielectrics generate remarkably high electrical fields, which may destroy the porous structure of the surface. Conversely, thick dielectrics (2 mm) may negatively affect the production of reactive species. Thus, at a gas gap of 5.5 mm and a dielectric thickness of 1.5 mm, the highest pore volume among the tested samples (0.2743 cm^3^ g^−1^) was measured by BET analysis and large pore volumes promote H_2_S catalytic oxidation. SEM analysis was also performed to evaluate the impact of the NH_3_ treatment on the surface morphology. A growth in the gas gap made the surface rougher while a negligible effect of the dielectric thickness on the structure was observed. In fact, increasing the gas gap increases the number of collisions between the reactive species and also favors the reaction between C, H and O atoms present on the catalyst surface, thus leading to CO_2_, H_2_ or H_2_O formation, which leave the surface in such high-energy environment.

**Figure 12:**
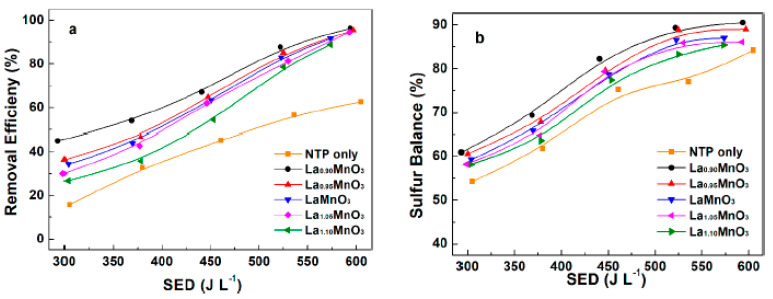
(**a**) H_2_S removal efficiency and (**b**) sulfur balance during hydrogen sulfide oxidation in a DBD reactor [74]; 10 kHz, 2 L min^−1^ of air containing 100 ppm of H_2_S, 1 atm, 80 °C.

Xuan et al. [74] studied the H_2_S removal over La_x_MnO_3_ perovskite catalysts in a DBD reactor. A coaxial type reactor, maintained at 80 °C, was used having a stainless road as high voltage electrode operating at a discharge frequency of 10 kHz; an air flow-rate of 2 L min^−1^ with an initial H_2_S concentration of 100 ppm was fed. The La_x_MnO_3_ (x = 0.9, 0.95, 1, 1.05 and 1.1) catalysts were prepared by the citric acid method and the effect of La content on the H_2_ removal efficiency was studied. The non-stoichiometric catalysts displayed lower surface area and smaller crystallite sizes compared to LaMnO_3_ catalyst and the best results were measured for the La_0.9_MnO_3_ sample, having an area of 15.2 m^2^ g^−1^ and a crystallite size of the LaMnO_3_ phase of 15.4 nm. The performance of the catalysts in the hybrid system as a function of the SED were compared with the H_2_S removal efficiency (i.e., conversion) recorded without the catalyst and the results are shown in Figure 12.

Whatever the selected La content, the presence of the catalyst enhanced the H_2_S removal and the sulfur balance compared to the performance of the NTP system without the catalyst. The stoichiometric catalyst displayed an increase in the H_2_S conversion as well as in the sulfur balance from 34.3% to 91.6% and 59.2% to 87%, respectively, with a growth of the SED in the interval 303.6–573.6 J L^−1^. The increase of sulfur balance with the SED was ascribed to the improved oxidation of the solid-state sulfur to SO_2_. In addition, among the non-stoichiometric samples, H_2_S conversion increased in the order La_0.90_MnO_3_ > La_0.95_MnO_3_ > La_1.05_MnO_3_ > La_1.10_MnO_3_ and the highest removal efficiency (96.4%) was recorded over the La_0.90_MnO_3_ at a SED of 593.7 J L^−1^. Thus, the best activity was measured for x = 0.9. Such result can be indeed related to the high surface area and the good dispersion of the LaMnO_3_ phase. In addition, the XPS study revealed that the latter sample assured the highest concentration of surface adsorbed oxygen, which displays higher mobility compared to the lattice oxygen. Therefore, the abundance of oxygen vacancies allowed enhanced oxidation rates of the H_2_S species.

Dang et al. [75] investigated the H_2_S removal in the presence of O_3_ over various metal oxide catalysts in a DBR reactor. The reactor, operating at 50 Hz, consisted of a tungsten discharge wire and a quartz cylindrical tube; a stainless-steel mesh, covering the reactor, acted as ground electrode. An air feed of 0.2 m^3^ h^−1^ with an initial concentration of H_2_S 200 mg·m^−3^ and 1200 mg·m^−3^ of O_3_ was sent to the reactor. The catalysts were coated on ceramic rings packed within the reactor. Then, 0.75 wt% of manganese, silver, copper and iron oxide were deposited on the rings via impregnation. The metal oxides were inactive for H_2_S removal in the absence of NTP and assured an improved hydrogen sulfide conversion compared to the case of the empty DBD reactor. The metal oxide activity increased in the order Mn > Ag > Cu > Fe: for an applied voltage of 22 kV, Mn_2_O_3_ reached complete H_2_S conversion while values of 98%, 82% and 75% were recorded over the Ag_2_O, CuO and Fe_2_O_3_ catalysts, respectively. The various oxides, in fact, displayed different ozone decomposition abilities, with the maximum quantity of decomposed ozone at 20 kV over the Mn_2_O_3._ In the H_2_S removal, ozone had a crucial role: its adsorption and/or decomposition on catalyst surface allows the formation of strongly oxidized species, which improves the H_2_S conversion. Thus, the Mn_2_O_3_ sample was selected for further studies. Three loadings (0.75 wt%, 3.85 wt% and 7.45 wt%) of the Mn_2_O_3_ catalyst on the ceramic rings were investigated, finding that the effect of the metal oxide content decreased with the applied voltage: at 12 kV, conversions of 42%, 81% and 89% were recorded for loadings 3.85 wt%, 0.75 wt% and 7.45 wt%, respectively. Conversely, at 22 kV, all the catalysts reached complete conversion. This result can be explained considering that the catalysts reach a fully activate stage only under high voltages, thus assuring enhanced H_2_S removal efficiency.

Table 4 summarizes the above results in terms of H_2_S conversion recorded during NTP in the presence of various catalysts.

### Conclusions

SO_2_ and H_2_S removal has been investigated over various NTP–catalyst-hybrid systems. In many cases, the dielectric barrier discharge reactor was selected while different catalysts as well as photocatalysts were selected. For sulfur dioxide removal, the selection of TiO_2_ was privileged while high H_2_S conversion was recorded in the presence of Alumina-supported catalysts. In this review, the effect of different parameters (including H_2_S as well as SO_2_ initial concentration, applied voltage, total flow-rate and discharge frequency) on the pollutant conversion was discussed, also highlighting the key role of catalyst properties (mainly active species dispersion) on hydrogen sulfide as well as sulfur dioxide removal efficiency.

## 5. NO_x_ Removal via NTP Technology

Nitrogen oxides (NO_x_), present in the environment in different forms such as NO, NO_2_, NO_3_, N_2_O, N_2_O_3_ and N_2_O_4_, are the main originator of photochemical smog and acid rains and represent a serious threat to the ecological environment and human health. The principal source of NO_x_ emissions is the combustion of fossil fuels, adopted in vehicles, incinerators, thermal power plants, etc., thus leading to a serious pollutant problem, present especially in developing countries [76].

**Figure 13:**
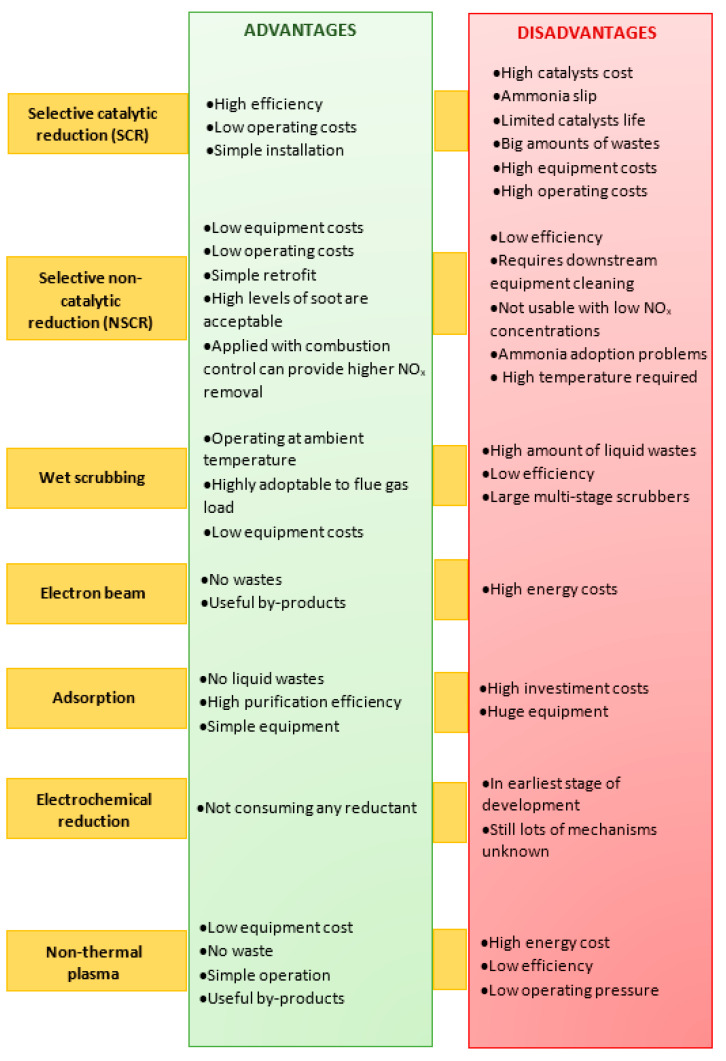
Advantages and disadvantages of the main NO_x_-removal technologies [77]. Adapted with permission from Fatemeh Gholami, Martin Tomas, Zahra Gholami, Mohammadtaghi Vakili, Science of The Total Environment; published by Elsevier, 2020.

The mainly adopted technologies in NO_x_ reduction are categorized by three different methods, namely, pre-combustion, combustion and post-combustion. While with pre-combustion and combustion methods, NO_x_ can be lowered by <50%, post-combustion techniques allow a >80% NO_x_ removal. Post combustion methods include selective catalytic reduction (SCR), selective non-catalytic reduction (SNCR), wet scrubbing, adsorption, electron beam, electrochemical reduction and nonthermal plasma (NTP), whose advantages and disadvantages are summarized in Figure 13 [77,78].

Among all the NO_x_-removal technologies, nonthermal plasma represents an interesting opportunity, as this method is used to selectively transfer energy to the electrons, thus avoiding the energy consumption necessary for the heating of the entire gas flow. Moreover, applying nonthermal plasma technology, many highly active oxidizing species (O·, ·OH, O_3_, etc.) can be generated, thus favoring the oxidation of NO to NO_2_ and the conversion of NO_x_ to nitric/nitrous acid, more easily eliminable compounds [79]. The most common NTP reactors applied for the NO_x_ removal are electron beam irradiation (EBI), dielectric barrier discharge (DBD) and corona discharge reactors (CDR) [77].

### 5.1. NO_x_ Catalytic Removal *via* NTP Technology

In the last years, several studies have been devoted to the coupling between the adoption of nonthermal plasma technology and the use of catalyst to lower the NO_x_ emissions originated by different sources; indeed, the pairing of the two technologies could also result in the enhancement of the catalytic activity and in a lowering of the catalyst activation temperature, as observed in a study concerning Ag/Al_2_O_3_ catalysts, adopted in the nonthermal plasma assisted hydrocarbon catalytic reduction (HC–SCR) deNO_x_ reaction. Hence, the authors found that, in comparison with conventional thermal activation, a strong enhancement of the catalytic activity was observed, characterized by high conversions of both NO_x_ and hydrocarbons at temperatures lower than 250 °C, at which the silver catalyst is normally not active. Moreover, a significant activity has also been registered in the absence of an external heat source, at temperature close to the ambient temperature, thus highlighting the possibility of developing vehicle exhaust treatment devices that could operate during cold start, that represent a serious problem in the field of pollutants emissions [80].

The activity of a similar catalyst (Ag/α-Al_2_O_3_) has been tested by Nguyen et al. [81] towards the HC–SCR of NO_x_ in a temperature range of 150–350 °C using a packed-bed dielectric barrier discharge plasma reactor. The study revealed that while with the HC–SCR system alone the NO_x_ reduction efficiency was in a range of 37.6% to 63.4% depending on the temperature, plasma coupling resulted in an enhancement of the reduction efficiency, achieving values of 74% in the whole temperature range; this result pointed out that the strong temperature dependence of the catalytic activity can be overcome by the addition of the plasma technology.

Wang et al. [82] tested a combined NO_x_ adsorption–CH4 plasma discharge process, suggesting that the plasma-assisted catalysis technology is one of the most efficient routes for deNO_x_. In their study a commercial synthetic H–MOR zeolite and a modified Co–ZSM-5 (Co–MOR) were tested at ambient temperature, giving a conversion of the adsorbed NO_x_ of 99.7% and 98.3%, respectively. Moreover, the authors also investigated the behavior as catalyst and adsorbent of one of the most common natural zeolites, characterized by abundant resources, the natural mordenite (NMOR), that is very economic and environmental-friendly. The co-modified NMOR shown higher NO_x_ adsorption capacity than the respective MOR, resulting in a NO_x_ conversion and N_2_ selectivity of 95% and 100%, respectively in the CH_4_ plasma discharge process. A further investigation concerning the capacity of NMOR for NO_x_ removal also highlighted the stability of the zeolite under cyclic operations.

Zhang and coworkers [83,84,85] performed a deep investigation on non-thermal plasma-assisted NOx storage and reduction (NSR) over Ba/Al lean NO_x_ trap (LNT) catalysts. These catalysts work cyclically, alternating their operation between two conditions, fuel-lean and fuel-rich; indeed, under lean exhaust conditions, characterized by high amount of oxygen, NO_x_ are adsorbed on the catalyst surface while, under rich conditions, NO_x_ reduction takes place as a consequence of the presence of an excess of reductant. Their first inspection concerned the comparison between two cobalt-containing catalysts (Co/Ba/Al and Pd/Co/Ba/Al) and the traditional Pt/Ba/Al catalyst in a H_2_–plasma assisted NSR process; when the H_2_–plasma was used in the rich phase to assist the reduction of the stored NO_x_, the Co/Ba/Al shown similar catalytic properties than those of Pt/Ba/Al. However, when there was the presence of H_2_O and CO_2_ in the feed, the NO_x_ removal efficiency was considerably lowered with these two latter catalysts, while in the case of Pd/Co/Ba/Al an excellent NO_x_ storage performance has been encountered by the authors, thus inhibiting the effect of the presence of H_2_O and CO_2_ in the feed [83]. Subsequently, their intent has been testing under the same process, non-thermal plasma-assisted NOx storage and reduction, Pt-free catalysts and, under this purpose, a series of M/Ba/Al (M = Mn, Fe, Co, Ni and Cu) catalysts have been prepared and tested by the authors. All the investigated catalysts shown higher activity in the oxidation of NO to NO_2_ and better NO_x_ storage capacity in comparison with the reference catalyst, Pt/Ba/Al; however, there were some difficulties in regenerating the NO_x_ sites during the rich phase, thus indicating that the rate limiting step for the transition metals catalyst is the regeneration of the stored NO_x_. Withal, addition of H_2_–plasma in the rich phase greatly improved the regeneration of the NO_x_-saturated sites, leading to an enhancement of the NO_x_ conversion, especially in the low temperature region [84]. Lately, their attention has been focused on the Pd/Co/Ba/Al sample and the comparison with Pd/Ba/Al and Pt/Ba/Al catalysts. The Pd–Co catalyst exhibited higher performances in both NO oxidation and NO_x_ storage capacity and, moreover, shown higher NO_x_ reduction activity with CO in comparison with the other two catalysts. Once again, the rate limiting step, has been identified in the regeneration of the stored NO_x_ in the low temperature region, thus leading to relatively low NO_x_ removal efficiency, especially below 250 °C and in the presence of CO. Furthermore, in this case, the employment of a plasma-assisted regeneration, adopting H_2_ or CO, led to an excellent NO_x_ removal efficiency in both lean and rich conditions, characterized by NO_x_ conversions of almost 99% in a temperature range of 150–350 °C, even when CO was used as the reducing agent [85].

LNT catalysts have also been investigated by Bai et al. [86], that analyzed the influence of transition metals doping on Pt/BaO/Al_2_O_3_ catalysts. In particular, the analysis performed on Pt/Ba/Al, Pt/Co/Ba/Al, Pt/Mn/Ba/Al, evidenced that, while the addition of Co and Mn led to improved NO_x_ storage capacity performances and consequently to superior cycle-averaged NO_x_ conversion, Cu doping has been found to worsen both NO_x_ storage efficiency and NO_x_ reduction activity, probably due to the formation of a Pt–Cu alloy. Better outcomes have been gained with the employment of Pt/Co/Ba/Al_2_O_3_, that exhibited NO_x_ conversions >80% in a temperature range of 150–350 °C and also shown the best SO_2_ resistance abilities.

Yu et al. [87] inspected the influence of the plasma carrier gas in a combined adsorption–discharge plasma catalytic process for the NO_x_ removal adopting zeolites as catalyst and in the absence of an external heating. The study pointed out that the conversion of the adsorbed NO_x_ was much lower in N_2_ plasma in comparison with an Ar plasma; the reason of such phenomenon has been attributed to the occurring of the NO_x_ formation reaction, indeed, the increase of oxygen species deriving by the decomposition of the adsorbed NO_x_ could represent the main cause of their collisions with nitrogen species, thus leading to the formation of NO_x_. Hence, in order to improve the NO_x_ removal efficiency, the authors doped the catalysts with solid carbon that could remove the active oxygen species, thus enhancing the conversion of the adsorbed NO_x_. The better outcomes have been reached for the H–ZSM-5 zeolite with the 8.5 wt% of solid carbon that allowed to obtain NO_x_ removal rate of 97.8%.

Another investigation concerning the combined adsorption–discharge plasma catalytic processes for the NO_x_ removal has been performed by Li et al. [88] through the preparation and testing of Cu modified carbon molecular sieves (CMS)-based catalysts. The authors inspected the NO_x_ adsorption on three different supports CMS, 13X zeolite and γ-Al_2_O_3_ finding that on CMS a higher NO_x_ adsorption and a lower desorption temperature have been achieved. Cu addition resulted in an enhancement of both NO_x_ adsorption and NTP removal capacity of CMS, indeed the NO_x_ removal capacity achieved was around the 96.2% over the 15% Cu–CMS catalyst in 30 min.

Pan et al. [89] evaluated the effects of NTP on the selective catalytic reduction of NO_x_ by CH_4_ (CH_4_–SCR) over a In/H–BEA zeolite in a wide range of reaction temperature (250–550 °C) noticing a beneficial effect derived by the NTP adoption especially at low temperatures, below 425 °C. Indeed, this improvement has been attributed to the promotion of the NO oxidation, while, at higher temperatures the synergetic effect gradually decreased. Moreover, from the catalysts characterization, it was also evidenced that the nonthermal plasma addition resulted in a promotion of the catalyst SO_2_ and H_2_O tolerance as a consequence of the inhibition of the SO_2_ oxidation, the InO^+^ sulfating and the indium oxide agglomeration.

Cui et al. [48] investigated an integrated system comprising a DBD reactor, a MnCu/Ti catalyst and a wet electrostatic precipitator for the simultaneous removal of NO and SO_2_. In the study, the effect of different initial concentrations of NO and SO_2_ was evaluated, showing that higher simultaneous removal of the two pollutants were obtained when the initial concentrations were the lowest. Moreover, a comparison between the DBD reactor alone and the coupling with the wet electrostatic precipitator pointed out that the coupling resulted in higher removal efficiencies and in particular, with low initial concentrations (SO_2_: 1000 mg m^−3^ and NO: 200 mg m^−3^) removal efficiencies of 93.4% for NO and 100% for SO_2_ have been obtained in the DBD-catalyst-wet electrostatic precipitator.

Wang et al. [56] evaluated the adsorption capacity of γ-Al_2_O_3_ under NTP technology for the simultaneous removal of NO_x_ and SO_2_, experiencing that a significant improvement in the NO_x_ adsorption on γ-Al_2_O_3_ was obtained in the presence of NTP. Indeed, in the presence of NTP, the ·O and O_3_ radicals would improve the oxidation of NO to NO_2_ and, to a lesser extent the oxidation of SO_2_ to SO_3_. Moreover, the interaction between the formed radicals and the γ-Al_2_O_3_ may produce O atoms adsorbed on adsorbents that could also be beneficial for the adsorption of NO_x_ and SO_2_ and could promote the formation of nitrate and sulfate.

### 5.2. NO_x_ Non-Catalytic Removal *via* NTP Technology

Numerous studies have been devoted in the last years to the investigation of NTP technology without the adoption of catalysts in the removal of air pollutants, indeed, the development of new types of nonthermal plasma technologies and eventual modifications to the previous systems have also attracted considerable attention in this field.

Kim et al. [90] through the evaluation on the repercussions of the nonthermal plasma application on the NO_x_ emissions in a model gas turbine combustor found that the presence of the flame improved the NTP generation and, at the same time, the NTP application contributed to the flame stabilization. As concern the NO_x_ emissions, a slight increase in their concentration has been encountered, due to a temperature rise, caused by the collision between neutron and populous electrons in the flame zone; however, their maximum concentration was around 10 ppm.

Paulauskas et al. [91] investigated the NO_x_ removal via direct plasma treatment from a real flue gas originated by methane combustion, finding that the residual oxygen plays a crucial role in the oxidation of NO to NO_2_ when oxygen concentrations are ≥6%. Moreover, when oxygen concentrations are below the 6%, the NO removal efficiency is increased because of other processes. From an energetic point of view, the plasma treatment for the NO removal shown a consumption that was only the 1% of the total generated power in the case that all the exhaust gas was fed through the plasma reactor.

Hashim et al. [92] tested a modular typed dielectric barrier discharge device for air remediation, experiencing an efficacy removal of NO_x_ from a gas stream containing high nitrogen oxides concentrations; in particular, with a six-tube DBD module, more than the 80% of nitric oxide has been removed from the gas stream.

Guo et al. [93] developed a new method in the field of exhaust gas purification by the combination of nonthermal plasma and wood fiber. From the comparison between the NTP alone and the combination with different wood fibers it has been seen that the addition of wood fibers increased the NO conversion efficiency, and the best outcomes have been obtained with the employment of scotch pine. Moreover, in their work, the influence of many operative parameters has been analyzed, giving as a result that (i) the increase of voltage could increase the NO_x_ removal, (ii) the increase of frequency could increase the NO_x_ removal, (iii) the increase of humidity promoted the NO_x_ removal, with a maximum at 25% humidity, indeed, after this value the conversion of NO decreased slightly.

Wang et al. [94] applied a DBD plasma with acetylene to a simulated thermal power plant and diesel exhaust noticing that a temperature increase led to an improved NO_x_ removal efficiency. The increase in the temperature favored the dissociation of C_2_H_2_, N_2_ and O_2_, thus improving the reaction rates of the reactions involved in the NO removal. Moreover, the authors observed that the addition of water at room temperature prevented the discharge in the DBD reactor and the ·OH radicals originated by the dissociation of H_2_O contributed to the reaction that produce HNO_3_ from NO_2_, thus favoring the NO_x_ removal efficiency.

**Figure 14:**
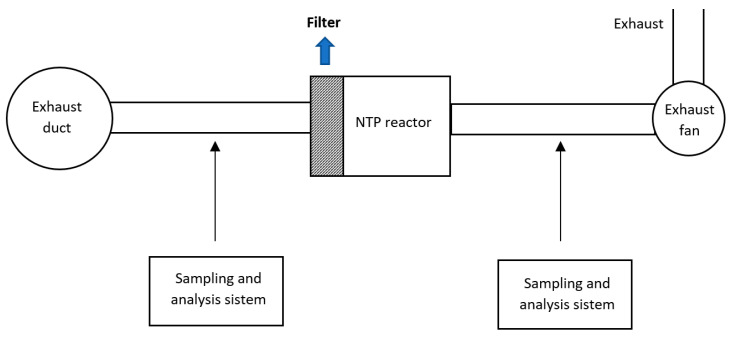
Scheme of the experimental non-thermal plasma (NTP) setup realized by Adnan et al. [95]. Reproduced with permission from Zulfam Adnan, Sadullah Mir, Mudassar Habib, Atmospheric Pollution Research; published by Elsevier, 2017.

Adnan et al. [95] realized important modification in the NTP technology to improve the depletion of pollutants in the exhaust gases coming from the fuel combustion. The authors developed a system comprising a filter that would allow to keep an adequate distance between two corona plates and a suitable gap between the electrodes and the barrier; moreover, the system would also make possible the changing in the gas flow rate through the NTP, operated by the modification of the revolution per minute (RPM) of the exhaust fans, as schematized in Figure 14:

It has been seen by the authors that the increase in RPM resulted in an enhancement of the NTP adsorption, thus leading to a NO_x_ reduction of more than 95%.

Yoon et al. [96] analyzed the simultaneous oxidation and absorption of NO_x_ and SO_2_ in an integrated O_3_ oxidation/wet atomizing system with the employment of a DBD reactor. The absorption of NO_x_ and SO_2_ was realized with a H_2_O_2_ solution supplied by an ultrasonic humidifier; H_2_O_2_ has been chosen by the authors as the most suitable oxidant due to the advantages gained from an economic and environmental point of view. NO_x_ and SO_2_ were absorbed as aqueous ions in the wet atomizing reactor that could allow to lower the gas–liquid contact time between liquid droplets and gaseous pollutants. The total removal efficiencies achieved in this study were 88.8% and 100% for NO_x_ and SO_2,_ respectively.

The simultaneous removal of NO_x_ and SO_2_ has also been investigated by Kang et al. [97] that performed a gas-phase oxidation with ozone and a wet scrubbing with sodium hydroxide; in their study the NO was oxidized to NO_2_ in a lab-scale reactor and subsequently the wet scrubbing with NaOH was conducted in a packed-bed scrubber. The NO_x_ removal tests have been performed with different SO_2_ inlet concentrations: (i) absence of SO_2_, (ii) SO_2_: 1000 ppm and (iii) SO_2_ > 1000 ppm.

(i) In the case of no SO_2_ at the reactor inlet, the ozone injection was the 60% with respect to the NO concentration and a total NO_x_ removal efficiency has been obtained, mainly attributed by the authors to the formation of trivalent N species (N_2_O_3_ and HNO_2_) and their fast absorption in NaOH;
(ii) With a SO_2_ concentration of 1000 ppm, the ozone concentration has been increased to the 90% of the NO concentration to improve the NO_x_ removal efficiency;
(iii) With higher SO_2_ concentration, even higher NO_x_ removal efficiencies were gained, because the SO_2_ scrubbing product, Na_2_SO_3_, improved the NO_2_ scrubbing.

The operating conditions and efficiency for NOx-removal via NTP technology are summarized in Table 5.

#### Conclusions

NO_x_ removal via NTP technology from the exhaust gas originated by different sources has attracted considerable attention in the last years due to the advantages gained with the employment of this technique. Both the nonthermal plasma technology alone and the coupling with the adoption of catalysts have led to obtain high NO_x_ removal efficiencies, with values that in most cases exceed the 80%. Moreover, it has been observed that, the coupling between the NTP technology and the catalyst adoption allows to obtain an enhancement of the catalytic activity and, in some cases, to lower the catalyst activation temperature, thus improving the processes efficiencies.

## 6. Soot Abatement via NTP Technology

Since the most increasingly problems connected to the diesel-soot emissions, several technologies have been proposed in the last years to allow the respect of the European Union regulations in this matter and, among them, the diesel particulate filters (DPFs), are nowadays, the most common after-treatment systems employed in the particulate matter (PM) trapping from the exhaust gases [98]. These systems usually operate in two steps, accumulation and regeneration, as described elsewhere [99], and the main challenges encountered in their on-board adoption regard the regeneration step. Indeed, during the DPF working the carbon deposition gradually blocks the filter, thus generating an increase of the exhaust backpressure and a decrease of the engine power. Hence, the performance of the DPF depends on the regeneration efficiency, and while the exhaust gas temperature is around 200–400 °C, the temperature necessary for the soot combustion should be around 550–600 °C [100]; on this purpose, several strategies have been proposed and adopted to afford the amount of energy necessary, including the fuel post-injection, the microwave adoption or the employment of catalysts to lower the soot initial combustion temperature [101].

NTP technology has been recently considered as an innovative approach in this field as, with its employment, the energy delivered to the system helps the electrons to accelerate and collide with the background molecules (N_2_, O_2_ and H_2_O), thus resulting in the production of secondary electrons, photons, ions and radicals that accelerate the soot oxidation reaction; moreover, the reaction between carbon and O atoms it has been estimated to be 40 times higher than that occurring in the presence of O_2_ [102,103].

The coupling between the NTP technology and the adoption of catalysts is an interesting issue in the PM removal; indeed, Ranji-Burachaloo et al. [102] suggested that the short-living reactive species formed in the discharge could play an important role in promoting the re-oxidation of metal oxide vacancies generated during the oxidation reactions in the discharge and, moreover, the catalytic decomposition of the ozone could also result in an increase of the energy efficiency and allow the removal of this harmful component from the outlet gas stream. In their study, under diesel exhaust gas conditions of 10% oxygen in a temperature range of 180–350 °C, an investigation concerning the soot-removal efficiency with the NTP technology alone and the coupling with different metal oxide catalysts has been performed. The soot-removal efficiency (SRE) has been defined by the authors as $\mathrm{SRE}=\frac{m\cdot 1000}{\mathrm{EI}\cdot t}\;\left[\frac{g}{\mathrm{kWh}}\right]$, in which m is the amount of removed soot [g], EI the energy injection [W] and t the time for the complete oxidation [h]. In absence of catalyst, varying the gas temperature and the EI (defined as EI = V·I, where V is the discharge voltage [V] and I the current [A]), the highest soot-removal efficiency has been obtained at 350 °C and 7.5 W, corresponding to a SRE of 5.04 g kWh^−1^. Subsequently, the authors investigated the adoption of Fe_2_O_3_, MnO_x_ and Co_3_O_4_ as catalysts in the corona plasma reactor, finding maximum values od SRE of 6.0, 7.0 and 5.6 g kWh^−1,^ respectively, at 350 °C and 7.4 W. The study pointed out that higher SRE were obtained with the catalysts adoption under the NTP system; indeed, the oxygen supply to the soot seemed to occur by reactive species generated in the plasma (i.e., O atoms, ·OH radicals and O_3_ molecules) and the catalyst through its contact points with soot. Moreover, the authors suggested that, in the presence of reactive species, with respect of molecular oxygen, the metal oxides oxygen vacancies generated by the soot oxidation are more easily refilled; thus, resulting in a synergistic effect of metal oxides on the soot oxidation promotion under plasma conditions.

Yao et al. [104] investigated a plasma–catalytic method for the removal of diesel PM in a low temperature range (100–250 °C), testing Au, Pt, Pd and Ag as catalyst for the soot abatement. A comparison with the plasma system alone has also been performed in the study and the result shown that, without the employment of a catalyst, after 1.1 h plasma processing, the PM removal was decreased due to the accumulation of soot within the plasma reactor. Among all the investigated catalysts, Au shown the best performances, characterized by a PM removal efficiency that did not decrease since the plasma switching off (8 h processing). Indeed, at the lowest temperature value, 100 °C, the PM removal efficiency obtained in absence of catalyst was 2.4 g kWh^−1^ and, with the adoption of Au as catalyst it increased to 4.0 g·(kWh)^−1^, while with the other catalysts (Pt, Pd and Ag) there were not significative improvement in the PM removal efficiency. At higher temperatures, 200 and 250 °C, the activity of the catalysts was in the order Au ≈Pt > Ag > Pd. A further investigation has been performed by the authors at 200 °C, with 20% O_2_ and P = 4.5 W for t = 1 h, to see the effect of the Au loading and, it has been seen that, the PM removal efficiency increased up to 6.1 g kWh^−1^ until the amount of Au reached 1 μg cm^−2^ and subsequently decreased as the deposited Au increased, maybe due to an Au particles agglomeration effect.

Zhang et al. [105] performed a O_3_ activation over two types of Ag/CeO_2_ catalysts, characterized by nanocube and nanorod-like morphologies, AgCe–C and AgCe–R, respectively. Both catalysts have been tested to see the effects of O_3_ activation on their soot oxidation performances; and the results shown that after the O_3_ activation at relatively low temperature, these catalyzed filters may combust soot effectively in exhaust environment. Indeed, during O_3_ activation, extra Ox-species were brought by O_3_ onto the catalysts surface, and these reactive species could result in a promotion of the soot catalytic combustion. Moreover, O^3^ activation led to structural changes that improved the soot oxidation activity of both catalysts. In particular, these phenomena were more pronounced in the case of AgCe–C that exhibited also high redox stability and good performances under different conditions.

**Figure 15:**
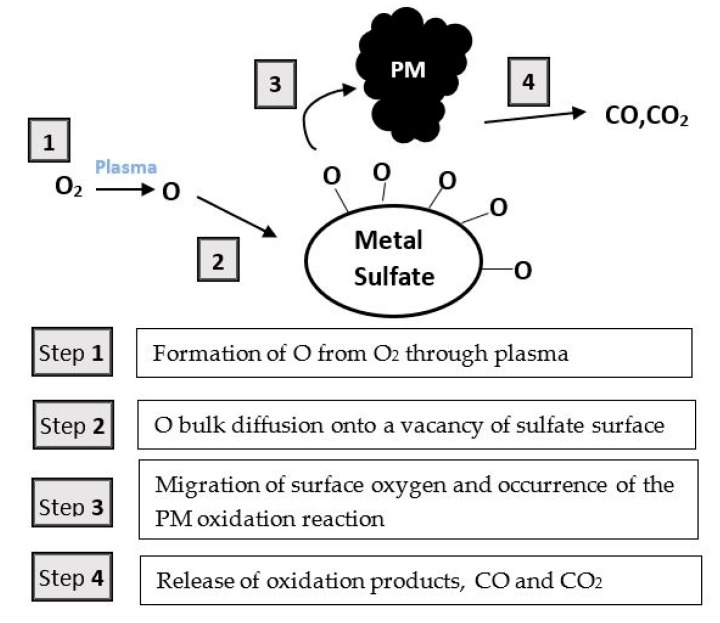
Mechanism of the plasma particulate matter (PM) oxidation enhanced by the metal sulfate proposed by Yao et al. [106].

Yao et al. [106] inspected the effect of the addition of metal sulfates in the nonthermal plasma induced oxidation of diesel particulate matter. Several metal sulfates have been tested by the authors: MgSO_4_, K_2_SO_4_, CaSO_4_·2H_2_O, Na_2_SO_4_, Fe_2_(SO_4_)_3_ and Al_2_(SO_4_)·8H_2_O and, among them, while with the first three an enhancement of the PM oxidation performances has been encountered, no significative effects were obtained with the latter three. The authors suggested that the advantages gained with the adoption of metal sulfates could be correlated to the adsorption of O atoms onto the sulfates and on this purpose, they also proposed a mechanism for the plasma PM oxidation enhanced by the metal sulfate, presented in Figure 15.

K_2_SO_4_ has shown in the research the better outcomes, with a soot-removal efficiency of 3.8 g kWh^−1^, obtained with the 5 wt% of the metal sulfate. The possible reasons connected to the K_2_SO_4_ catalytic effect were summarized by the authors as (i) The K atoms could increase the amount of chemisorbed oxygen on the catalyst surface, thus improving the energy yield, (ii) K atoms could enhance the catalysts activity by forming eutectic compounds, (iii) K atoms could improve the PM oxidation by enhancing the carbon consumption into the formation of carbon intermediates, (iv) free K atoms are the active species in potassium-enriched catalysts for the PM combustion, and the strict contact between the PM and K cations allows to lower the soot combustion temperatures.

Nguyen et al. [107] investigated a HC–SCR system for the simultaneous removal of NO_x_ and soot on Ag/α-Al_2_O_3_ catalyst. In particular, the investigation concerning the soot abatement has been performed by the authors by adopting a soot simulant, naphthalene. In the study, the simultaneous removal of NO_x_ and soot has been examined in a temperature range of 150–350 °C and by varying the SEI in the range up to 210 J L^−1^.

**Figure 16:**
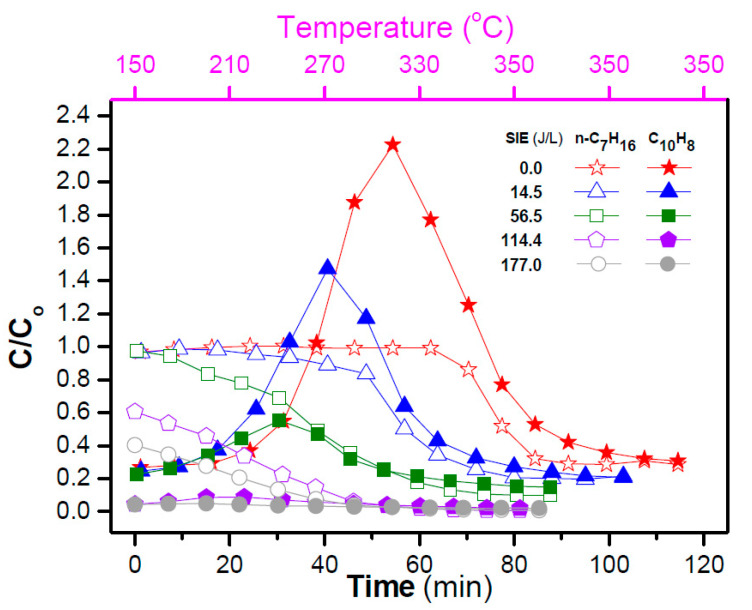
Evolution of the concentration ratio (C/C_0_) of naphthalene and n-heptane as a function of temperature and time [107].

The study outcomes are presented in Figure 16, in which the ratio of the concentration at the outlet and at the inlet (C/C_0_) for both naphthalene and n-heptane is depicted as a function of the temperature (from 150 to 350 °C with a heating ramp of 3 °C min^−1^) and as a function of the elapsed time for various SEI values. The first concentration increase exhibited in the case of naphthalene has been attributed to the desorption occurring at high operating temperatures and moreover, it has been seen that, the maximum value reached in terms of naphthalene concentration decreased as the SEI increased, thus indicating the result of the naphthalene decomposition by the plasma action. The advantages of the plasma action were also identified as concern the C/C_0_ of naphthalene; indeed, the outlet concentration largely decreased with the adoption of plasma. As regard n-heptane, no maximum in the concentration profiles were detected, in fact, the C/C_0_ of n-heptane decreased as the SEI and operating temperature increased. The difference in the behavior of naphthalene and n-heptane has been attributed by the authors to their adsorption capabilities and vapor pressures, as the n-heptane vapor pressure is greater than that of naphthalene.

The operating conditions and soot-removal efficiency via NTP technology are summarized in Table 6.

### Conclusions

The coupling between the NTP technology and the adoption of catalysts is a deeper investigated approach for the soot abatement in the diesel exhaust gas, as it has been demonstrated that it could bring several benefits in terms of soot-removal efficiency. In this manuscript, an overview on the latest NTP applications in this field has been presented, giving a comparison between metals, metal oxides and metal sulfates as catalysts for the PM removal.

## 7. CO_2_ Utilization

A big scientific and industrial interest has been devoted in the last year to environmental pollution. As it is well known, the emission of greenhouse gasses, in particular CO_2_, must be mitigated and among the possible solutions which have been proposed the carbon capture, storage and utilization (CCSU) technologies have widely spread. non-thermal plasma (NTP) is a promising technology for CO_2_ conversion; in fact, it is well-known that the breakage of C=O bond requires a huge energy amount: plasma allows the occurrence of thermodynamically unfavorable chemical reaction at ambient condition—and this is the case of CO_2_ dissociation [108]. NTP usually works within the electron energy range of 1–10 eV: the electron energy value of 5.5 eV is sufficient to provide C=O bond breakage via stepwise vibrational excitation. Different routes for CO_2_ conversion have been investigated, and, in particular, CO_2_ conversion associated with NTP application deals principally with CO_2_ reforming of methane, also called dry reforming of methane (DRM) and CO_2_ hydrogenation to value-added fuels and chemicals.

Conventional hydrocarbon conversion processes still represent the most widely employed technologies in the chemical industry: the most representative may be considered methane reforming (MR), as it is the most promising technology used to date for hydrogen supply. Nevertheless, as it is well-known, MR is a high energy-consuming process and, furthermore, a remarkable amount of energy is actually lost during the process. To better define this consideration, authors have proposed the concept of exergy, as the maximum work available via a change of state. In methane steam reforming (MSR), 30% of initial exergy is destructed through the combustion of a fraction of methane to supply the energy required from the furnace; another exergy huge destruction occur in the water-to-steam heat exchange [109].

In the forthcoming energy scenario, energy supply to reacting system may not occur as heat addition (thermal energy) anymore, but conversely it may be provided as electrical energy. To date, hydrocarbon conversion in electrical energy-assisted processes is widely discussed and investigated. A promising technology is represented by nonthermal plasma, which has the double effect of providing energy to the system and generating radical and excited species, which in turn allow the initiation and the proceeding of the chemical reactions. Several discharge methods allow the generation of nonthermal plasma, i.e., gliding arc, microwave, corona discharge, dielectric barrier discharge (DBD) and others [110]. Reforming of methane, diesel, gasoline and propane by DBD have been widely reported in literature; among the innovative proposals to perform hydrocarbons reforming in a more efficient way, plasma systems could provide interesting solutions in terms of compactness, reactivity and efficiency of the overall process [111].

### 7.1. CO_2_ Reforming of Methane

**Figure 17:**
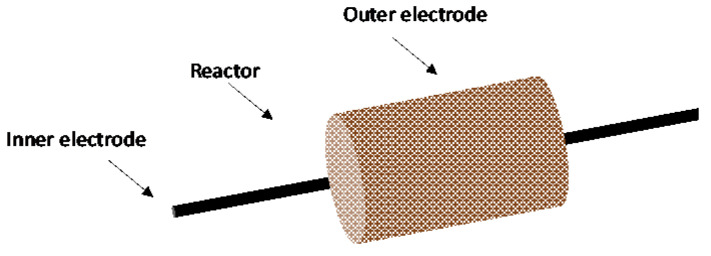
Schematic representation of a DBD plasma reactor.

As aforementioned, methane dry reforming has attracted a wide interest in the last years, because it actually represents a challenging method for the utilization of the two main greenhouse gasses, namely methane and carbon dioxide and their conversion into valuable fuels. The process suffers of several drawbacks, and the most important is the huge energy amount required for the reaction to occur. For the development of an efficient DRM process several technologies have been evaluated and, among them, plasma processes have acquired increasingly importance. In particular, the scientific word focused on dielectric barrier discharge (DBD) cold plasma technology (Figure 17), as it presents easy upscaling opportunities and mild operating conditions [112]. DBD, also called silent discharge, is the electrical discharge that occurs between two electrodes in a planar or, more often, cylindrical configuration (Figure 1); the two electrodes are separated by an insulator that is called dielectric barrier [113].

Generally, methane dry reforming is performed as catalyzed process with conventional heating. In presence of plasma, the ionization of the gaseous stream allows the spontaneous CH_4_ and CO_2_ dissociation: this leads to their conversion, but the selectivity of the process is relatively low, as several byproducts are produced, including coke, ethane, acetylene and other hydrocarbons. This drawback of course limits the nonthermal plasma application to the reaction. The combination of NTP technology with catalysts offers, instead, the possibility to have an easier breakage of the reactant molecules—together with a higher selectivity towards hydrogen and carbon monoxide formation. There are three ways to combine plasma and catalysis: (i) to perform a pretreatment of the catalyst with NTP, as it may change physicochemical characteristic of the catalyst, (ii) to use a two-stage system, in which the gas flows through the plasma region and then, once the reactive species are formed, it can encounter the catalyst and (iii) to combine the effects in a single-stage hybrid system, which is also called in-plasma catalysis (IPC), where the plasma region and the catalytic bed are overlapped [114].

#### 7.1.1. Catalytic NTP-Assisted CO_2_ Reforming of Methane

Among the possible combination between catalysis and plasma, the more employed configuration in the experimental study is the single-stage hybrid system.

Lu et al. [115] investigated the CO_2_ reforming of methane in a coaxial DBD plasma reactor evaluating the effect of nonthermal plasma and the effect of plasma-assisted catalytic activation on the reaction. The catalyst selected for the study was an innovative g–C_3_N_4_ (graphitic carbon nitride)-based formulation, to which TiO_2_ was added in different amount in order to also evaluate the influence of loading. The authors observed that, in the pure plasma process, the reaction performances were strongly affected by the input power, the total flowrate and the CH_4_/CO_2_ molar ratio. As expected, low gas flowrate and high input power allowed the achievement of the highest conversion values, even if the increase in power led to a decrease in the selectivity of the process. The presence of the TiO_2_/g–C_3_N_4_ catalyst allowed the enhancement of the catalytic performances only for low TiO_2_ loadings, in particular the 1% TiO_2_/g–C_3_N_4_ formulation gave the highest conversion and yield values. The authors explained these results by associating the higher TiO_2_ loading to a higher energy electrons adsorption, which resulted in reducing the reaction probability between high energy electrons and catalyst.

Khoja et al. [116] studied the CO_2_ reforming of methane in a DBD plasma reactor in presence of a 10% Ni/γAl_2_O_3_–MgO catalyst, prepared by wetness impregnation method assisted by cold plasma treatment. Plasma assisted catalytic DRM gave better results than pure plasma DRM, attesting the synergy between plasma and catalyst, who also shown a high resistance to coke formation. Ni loading was studied as a process parameter, and the authors found an optimum loading in the range 10%–20%: both the formulations allowed the achievement of very high conversion values, respectively around 75% for methane and 74% for CO_2_. In a subsequent work, the authors evaluated the performance of a 10% Ni/La_2_O_3_–MgAl_2_O_4_ for methane dry reforming in a non-thermal plasma DBD reactor [117]. The incorporation of La_2_O_3_ as co-support into MgAl_2_O_4_ was to change the irregular structure of MgAl_2_O_4_ into flakes, and it also enhanced the interactions among Ni and the support and the basicity of the catalyst. A comparison between the pure plasma reaction and the catalyzed plasma-assisted reaction shown that the catalyst presence remarkably improved the reaction performances, in terms of conversion of both reactants and of H_2_/CO ratio. The formation of La_2_O_2_CO_3_ as reaction intermediate, due to CO_2_ chemisorption, was found to help La_2_O_3_ regeneration and, consequently, to improve the catalyst stability. Later, the same catalyst (La_2_O_3_ co-supported Ni/MgAl_2_O_4_) was deeply studied in order to evaluate the role of several process parameters, such as of SEI, discharge volume and GHSV [118]. The systematic study of the variation of these parameters led to the development of a modified power-law model, where the rate expressions were defined as follows:

(18) $$k_{{\mathrm{CH}}_{4}}\left(J^{-1}{\mathrm{atm}}^{-1}\right)=\left[\frac{X_{{\mathrm{CH}}_{4}}}{1-X_{{\mathrm{CH}}_{4}}}\cdot \frac{1}{\left(\mathrm{SIE}\right)\cdot V_{D}\cdot p}\right]$$

(19) $$k_{{\mathrm{CO}}_{2}}\left(J^{-1}{\mathrm{atm}}^{-1}\right)=\left[\frac{X_{{\mathrm{CO}}_{2}}}{1-X_{{\mathrm{CO}}_{2}}}\cdot \frac{1}{\left(\mathrm{SIE}\right)\cdot V_{D}\cdot p}\right]$$

As result, the authors observed a linear function of SEI and GHSV against the instantaneous conversion of the reactants; the model was well representative of the experimental data and gave as activation energy values 32.6 kJ mol^−1^ for CH_4_ and 35.2 kJ mol^−1^ for CO_2_.

Mahammadunnisa et al. [119] investigated the plasma-assisted DRM in presence of a Ni/Al_2_O_3_ catalyst with different Ni loadings. The selected technology was the DBD plasma reactor, realized with a stainless-steel inner electrode and a copper wire wrapped at the external wall of the quartz reactor as outer electrode. The pure plasma condition was tested, but with the addition of a catalyst to this system it was observed an enhancement in the reaction performances: in particular, 20% Ni/Al_2_O_3_ gave the best results and allowed to achieve a 2.25 H_2_/CO ratio, which was remarkably higher than the 1.2 ratio obtained in the pure plasma condition. The authors ascribed the better catalytic behavior of the cited sample to the formation of nanocrystalline NiAl_2_O_4_, highly dispersed Ni nanoparticles and higher reducibility of the sample. In fact, spinel formation enhanced the particle dispersion resulting in lower particle size for the 20% Ni/Al_2_O_3_ sample (<6 nm) if compared to 10% Ni and 30% Ni samples, where Ni particles sizes were 13 and 18 nm, respectively.

Chung et al. [120] investigated the dry reforming of methane in a DBD plasma reactor in which three different packing materials were loaded: two ferroelectrics materials, BaZr_0.75_T_0.25_O_3_ (BZT; εr = 149) and BaFe_0.5_Nb_0.5_O_3_ (BFN; εr = 2025) and glass beads (r = 3–5). The study pointed out that, with plasma alone, the higher the ratio CH_4_/CO_2_ the higher was the reactants conversion and that it is possible to operate the system with high flowrates, which allow to generate syngas in a more economical way because of the better energy efficiency. The main result of the study was the adoption of packing relaxor ferroelectrics to enhance microdischarges with the aim of improving the DRM performances. This phenomenon was actually observed and the increased density in microdischarges produced much more collisions between high-energy electrons and gas molecules. Among the ferroelectrics materials, BFN packed bed ensured the better performances at any applied voltage, both in terms of conversion and selectivity, while the glass bead packed bed produced even worse results than plasma alone.

Mei et al. [121] studied the biogas reforming in presence of a Ni/γ–Al_2_O_3_ catalyst with various Ni loadings in a DBD nonthermal plasma reactor. As result, they observed that the parameter which had the highest influence on the hybrid plasma–catalytic reaction system was the biogas flowrate, while the CO_2_/CH_4_ molar ratio was the most significant factor affecting the energy efficiency of the process. The best results were achieved in the following conditions: biogas flowrate of 56.1 mL min^−1^, input power of 60 W, CO_2_/CH_4_ molar ratio of 1.03 and Ni loading of 9.5%. In particular, the optimal Ni loading was slightly influenced by other process parameters, so that in different conditions were found different optimal Ni loadings.

Yap et al. [122] investigated the methane and CO_2_ conversion into syngas through a plasma discharge coupled with a catalyst. The selected catalyst was 10% La_2_O_3_/alumina balls: considering that at ambient temperature the catalyst was inactive, the experimental tests were conducted both at ambient temperature and at 300 °C; other tests were performed using glass spheres as inert packing. As result, the authors reported that at room temperature only a slight difference was observed between the pure plasma and the catalyzed system, whereas at 300 °C the synergy between plasma and catalyst was clearly demonstrated. Furthermore, at 300 °C the catalyzed system achieved a CH_4_ conversion 3 times higher than CH_4_ conversion reached in the glass spheres system. Another important observation was that CH_4_ conversion was remarkably enhanced by the catalyst presence, while it was not the same for CO_2_ conversion. The authors proposed that CO_2_ dissociates, forming oxygen species in gas phase: these replenish the surface oxygen species which react with methane, according to Mars and Van Krevelen mechanism.

Wang et al. [123] investigated the DRM under pure plasma, catalyst only and plasma assisted with catalyst conditions, in order to determine the synergistic effect of plasma and catalyst. As catalyst, they selected Ni/AC (activated carbon) and several samples were tested varying the reduction temperature from 400 to 900 °C. The sample calcined at 700 °C (Ni/AC700) shown the highest specific surface area and highest pore volume, and it was selected as the most promising catalyst, as it reached CO_2_ and CH_4_ conversions, respectively of 64.6% and 65.7%.

Nguyen et al. [124] studied the DRM reaction in a DBD plasma reactor coupled with a Ni/α-Al_2_O_3_ catalyst in a peculiar configuration in which the ground electrode was covered with an insulating oil jacket. This was to prevent external power dissipation, which could lead to both loss of energy for the endothermic reaction and air discharge which give rise to the formation of harmful substances, for example NO_x_. The study pointed out that with the normal reactor configuration there was generation of plasma at the surroundings of ground electrode, forming NO_x_ and low conversion of methane and carbon dioxide because of the partial loss of energy: these issues were increasing evident at high input power. The immersion of the ground electrode in an insulation oil concentrated the discharge energy into the reaction zone, thus enhancing the overall conversion and selectivity values; furthermore, no NO_x_ formation was detected as side effect.

Wang et al. [125] studied the dry reforming of CH_4_ and CO_2_ on a Ni/C catalyst promoted by Ce, varying the Ce content obtaining a formulation NiCe_x_C where x = 0, 0.5%, 1%, 3%, 5%. The catalytic activity was evaluated in an NTP–DBD reactor. Characterization results shown that there was a strong metal–support interaction between Ni and ceria and that the samples presented a decreasing Ni particle size when Ce loading was increased, reaching values ranging from 31.1 (x = 0) to 23.7 (x = 5). Despite in general Ce promotion of Ni/C catalyst gave a satisfactory result, the most promising formulation was NiCe_1_C, reaching CO_2_ and CH_4_ conversion of 53.7% and 55.6% and H_2_ and CO selectivity of 50% and 53.2%.

Vakili et al. [126] reported that the shape of the packing material can influence the DRM because of the different geometries induce different microdischarges: in particular, it was observed that ZrO_2_ suppressed the plasma generation, while UiO-67 (a class of zirconium-based MOFs [127]) enhanced the reaction performances, as it provokes the generation of filamentary microdischarges and surface discharges thanks to its porous nature. UiO-67 slightly increased the reactants conversion and the selectivity to H_2_ and CO, while it dramatically modified the byproducts distribution, in particular it decreased the selectivity to C_2_H_6_ and C_3_H_y_ and increased the selectivity to C_2_H_2_/C_2_H_4_. In addition, also a 2% PtNP@UiO-67 catalyst was prepared (where PtNP denotes platinum nanoparticles) and it was found to improve the plasma-assisted DRM, as Pt presence enhanced the surface reactions: in particular, this sample allowed the achievement of the highest H_2_ selectivity and also the highest H_2_/CO ratio. Under conventional thermal activation, 2% PtNP@UiO-67 catalyst shown extremely low conversion value (<10%) demonstrating that plasma had a remarkable effect on the catalytic activity. A stability test pointed out that 2% PtNP@UiO-67 strongly decreased the selectivity to lighter hydrocarbons, thanks to the dehydrogenation of C_2_H_6_ and C_2_H_4_ over PtNP. Furthermore, the catalyst also demonstrated a high stability overtime in several on–off plasma cycles, showing no modification in the morphology and crystallinity and a non-appreciable sintering of PtNP, which were found to be around 1–4 nm, as a consequence of the tests.

Zheng et al. [128] reported a study on the synergistic effect of a LaNiO_3_@SiO_2_ NP catalyst and nonthermal plasma in a DBD reactor on the reforming of CH_4_ and CO_2_. The catalyst was prepared via the modified Stöber method [129] in order to realize a shell-core structure: this was supposed to provide a dedicated space for excited species and metallic actives accommodation. Furthermore, the particular support structure ensured a uniform dispersion of the fine nickel particles. Compared to pure-plasma and other catalytic systems (Ni/SiO_2_, Ni/La_2_O_3_ and Ni–La_2_O_3_/SiO_2_), plasma combined with the LaNiO_3_@SiO_2_ NP sample shown enhanced catalytic performances in all the evaluated test conditions. Furthermore, the competition of internal mass transport with the chemical reaction was evaluated through the Koros–Nowak criterion, and it was observed that the former was negligible. For this reason, a semi-empirical power-law kinetic model was developed, and the obtained apparent activation energy values were 24.73 kJ mol^−1^ for CH_4_ and 29.50 kJ mol^−1^ for CO_2_, which were relatively low if compared to the literature values: this was ascribed to the high dispersion of Ni on the shell-core support and to the strong core/shell interactions.

Bouchoul et al. [130] tested three different calcium-based materials for CO_2_/CH_4_ reforming in a DBD plasma reactor with and without the addition of water; the chosen input power (8 W) excluded the thermal activation of reactants. It was observed that reactants conversion was not significantly influenced by the nature of the Ca-based material, and the only detected difference was a lower formation of formaldehyde in presence of CaO. For this reason, this sample was further studied, and in particular it was shown that the increase in temperature from 100 to 300 °C slightly enhanced the methane conversion. When water was added to the feed stream, the catalyst underwent a deep modification, which involved hydroxylation for CaO and carbonation in the case of Ca(OH)_2_.

Mustafa et al. [131] studied the methane conversion in inert atmosphere in a double dielectric barrier discharge (DDBD) plasma reactor. CH_4_ concentration of 1000, 5000, 10,000 ppm where employed and two different configurations with a plasma discharge gap of six millimeters and three millimeters were selected. A comparison between the plasma–catalyst DDBD system and the pure plasma DDBD system shown that the former configuration gave the best results in terms of methane conversion, with a maximum value achieved of 84.93%; moreover, the hybrid solution also provided a higher energy efficiency than pure plasma system. Among the catalyst studied, Pt–Sn/Al_2_O_3_ gave the highest conversion efficiency in the whole range of input power investigated. The three millimeters discharge gap was found to be preferential in order to enhance methane conversion and, in general, the system was found to be suitable for low methane concentration, thus the authors recommended the technology to landfill at the “after care” stage.

#### 7.1.2. Non Catalytic NTP-Assisted CO_2_ Reforming of Methane

Tan et al. [132] evaluated the CO_2_ reforming of methane by the study of a simulated biogas conversion in a plasma-assisted reformer. The technology the authors chose was a gliding arc plasma and the reaction was performed in a pure plasma condition, i.e., in absence of a catalyst. The study pointed out that with the increase in input voltage, the reactants conversion increased; furthermore, also a longer exposure time to plasma led to higher conversion values, as it determined the production of more high-energy electrons, ions and radicals which enhanced the reactivity of biogas. Of course, both the increase in reaction time and in input power obviously means a consequential increase in the total consumption of electrical energy, thus both these parameters must be opportunely optimized in order to save energy.

Martin-del-Campo et al. [133] investigated the CO_2_ reforming of methane in a rotating gliding arc (RGA) nonthermal plasma reactor in a pure-plasma condition. The most efficient operating condition was developed by studying the effect of different parameters, in particular peak arc current, total gas flowrate, feed ratio and gas inlet preheating. As result, it was observed that doubling the peak current allow to increase the reactants conversion, but it had no significant effect on the H_2_/CO ratio. The increase of the feed flowrate also seemed to be beneficial, as it produced an increase in the arc length which enhanced the conversion. As expected, the higher the CO_2_ concentration in the feed gas (high CO_2_:CH_4_ ratios) the lower was the H_2_:CO ratio in the products stream.

##### Conclusions

CO_2_ reforming of methane was deeply studied both in pure plasma and in plasma-catalyzed system. As a common result, the hybrid configuration gives better reaction performances, in terms of activity, selectivity and stability of the catalyst. Among the possible catalytic formulations, Ni is the most encountered active metal, while Mg–Al oxides are extensively employed as support; lanthana-doped catalysts are also widely studied for this application. Furthermore, several studies pointed out the remarkable influence that a particular catalyst shape could have on the process, as a change in the microdischarge type can enhance the influence of plasma on the reacting mixture. A summary of the research article reviewed in sub-Section 7.1 is provided in Table 7.

### 7.2. CO_2_ Reforming of Methane

CO_2_ hydrogenation to methane, also called CO_2_ methanation or Sabatier reaction, is a promising technology to convert carbon dioxide coming from industrial processes or carbon capture and storage (CCS) systems in methane, which is a valuable fuel. Furthermore, CO_2_ methanation is the core step of the power-to-gas processes, in which surplus electrical energy coming from renewable sources is converted in hydrogen via water electrolysis and then in methane via the Sabatier reaction (Equation (20)).

(20) $${\mathrm{CO}}_{2}+4H_{2}⇄{\mathrm{CH}}_{4}+2H_{2}O$$

**Figure 18:**
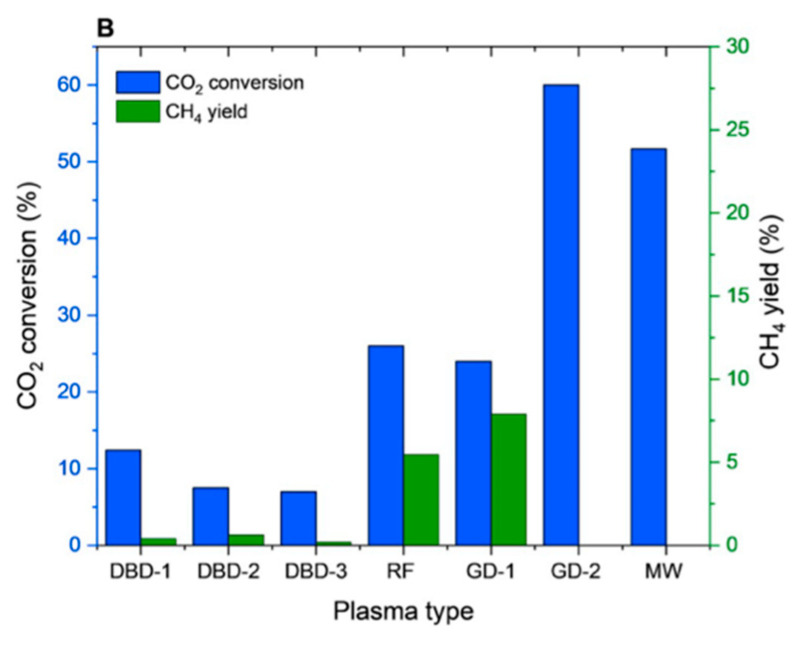
CO_2_ methanation in plasma-assisted processes without the presence of catalyst [136]. Adapted with permission from Radosław Dębek, Federico Azzolina-Jury, Arnaud Travert, Françoise Maugé, Renewable and Sustainable Energy Reviews; published by Elsevier, 2019.

It is clear why the CO_2_ methanation has acquired so much interest in the last years, but the scientific world is still facing the main constraint of the process: the high exothermicity of the reaction provokes a remarkable temperature increase in the catalytic bed, thus limiting the conversion and damaging the catalyst. In order to offer a solution to this problem, several possibilities have been experienced from the catalysis point of view: from low-temperature innovative catalytic formulations [134] to high conductive structured catalysts [135]. non-thermal plasma actually represent a very interesting technology to perform this reaction, as it offers the possibility to activate carbon dioxide at very low temperature and to modify the catalysts surface, giving rise to the creation of defects, surface vacancies and modification of surface functional groups, which may enhance the reaction extent and open new reaction pathways. Among the type of non-thermal plasma employed for CO_2_ methanation, the most encountered are for sure DBD, gliding arc (GA) and microwaves (MW). In the extensive review of the plasma-assisted CO_2_ methanation by Debek et al. [136] an interesting comparison of the efficiency of these technologies has been proposed: DBD offers the lowest conversion values, while the highest values are reached by MW and glow discharge (GD). What clearly emerges from this study, however, is that these processes are not selective, as the methane conversion is basically null, when they are conducted without presence of a catalyst (Figure 18).

For what concerns, instead, the hybrid plasma–catalytic CO_2_ methanation, several catalytic formulations have been investigated, pointing out two important aspects. At first, active metals in thermal CO_2_ methanation are active as well in the plasma-assisted process and the influence of the active specie is almost the same than in thermal process. Furthermore, Ni represent a consolidated active phase for CO_2_ methanation catalysts, as it has good activity and selectivity widely proved in the thermal process: for the plasma-assisted reaction, this result is still confirmed. An overview of the Ni-based catalysts tested under plasma condition for CO_2_ methanation up to 2019 has been reported yet [28]: this paragraph aims to update the previous results with the newest research achievements, in view of the high interest of this topic.

Sivachandiran et al. [137] studied a Ni/γ-Al_2_O_3_ nanocatalyst in the DBD plasma-assisted CO_2_ methanation. The authors evaluated the effect of temperature and input power on the reaction performances, in terms of CO_2_ conversion and CH_4_ selectivity. The study compared the role of the catalyst in the hybrid configuration with its role in thermal process; moreover, the effect of support (in thermal and plasma process) and the effect of plasma alone were evaluated and compared. From the experimental results, it was possible to observe that CO_2_ methanation did not proceeded in thermal mode without either catalyst or support, while it was possible to appreciate a slight CO_2_ conversion in plasma mode; moreover, in presence of the support a low value of CO_2_ conversion was obtained both in thermal and plasma mode, with the latter giving a better result (methane selectivity was not reported in any case). Nevertheless, the highest conversion value achieved was 2% (Al_2_O_3_ + plasma), so again it was confirmed that CO_2_ methanation reaction dramatically needs a catalyst to occur. The comparison of the catalyst activity in thermal mode and the reaction performances in the hybrid plasma–catalyst configuration pointed out that plasma allowed an earlier catalyst activation (the systems reached the same conversion value with a temperature shift of 50 degrees) and at 250 °C, where the catalyst is only active under plasma, a selectivity to methane of 70% and to CO of 3% were reached. The authors ascribed this result to the plasma-assisted in situ reduction of Ni^2+^/Ni. Nevertheless, above 300 °C in the plasma-assisted catalyzed system the methane reforming was promoted, as CO selectivity was observed to increase up to 7%; moreover, at high temperature, an increase in plasma input power further increased CO selectivity. For these reasons, the system was particularly promising for low-temperature applications; furthermore, Ni nanocatalyst was not structurally affected by plasma application, as the Ni particle size was not found to increase significantly (from 14 to 15 nm).

**Figure 19:**
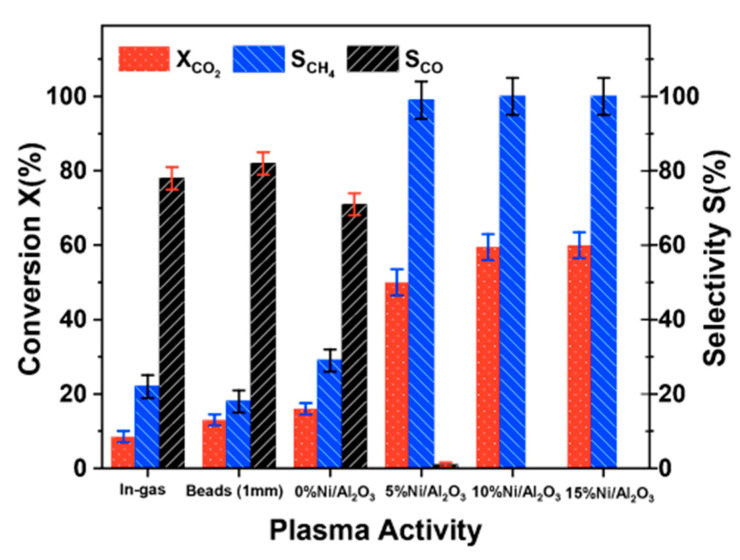
CO_2_ methanation performance in plasma-assisted process with different operating modes from [138]. Adapted with permission from Farhan Ahmad, Emma C. Lovell, Hassan Masood, et al., ACS Sustainable Chemistry & Engineering; published by American Chemical Society, 2020.

Ahmad et al. [138] investigated the performance of three Ni/γ-Al_2_O_3_ catalysts with different Ni loadings (5%, 10% and 15% wt) in plasma-assisted CO_2_ methanation in a DBD packed bed reactor. Thermal and plasma-assisted catalytic tests were performed evaluating CO_2_ conversion and CH_4_ selectivity. From the thermal activity tests, it was possible to observe the positive effect in increasing the Ni loading from 5% to 10%, while 10%- and 15%-Ni samples shown the same conversion and selectivity trend; this result was obtained also under plasma application. In the plasma-assisted process, also the effect of the bare support, inert beads and plasma alone were evaluated, pointing out that in the absence of catalyst the main reaction product was CO, with only a 20%–30% selectivity to methane, while CO selectivity in presence of any catalyst was almost zero, as reported in Figure 19. The authors attributed the high performances of the plasma–catalytic system to plasma activation of the reactants, to the presence of Ni (which was not detrimentally affected by plasma discharges) and to the synergy between catalyst and plasma-activated reactants.

Wang et al. [139] focused on the evaluation of the performances of CO_2_ methanation in plasma-assisted catalyzed reaction in presence of a Ni/Ce_0.58_Zr_0.42_O_2_ catalyst. The study compared the reaction in a thermal system and in a DBD reactor and the steady-state temperature of the reaction was evaluated through experimental and simulation analysis with the software COMSOL Multiphysics^®^. The main objective of the study was the estimation of the threshold temperature in the plasma-assisted configuration: for this reason, in order to minimize the effect of the heat generation from the reaction, a very low inlet flowrate was employed. Coupling the experimental results with the simulation analysis the authors obtained that the plasma threshold temperature for CO_2_ methanation was 116 °C.

Chen et al. [140] studied the Sabatier reaction in a DBD plasma reactor in presence of Ni supported on metal–organic frameworks (MOFs). The results highlighted the advantage in using MOFs as support rather than conventional ZrO_2_ and that the activation barrier for the plasma-activated reaction was remarkably lower than for the thermal catalysts, in particular about 32 kJ mol^−1^ for the plasma-assisted process and about 70 kJ mol^−1^ for the thermal process.

Biset-Peiró et al. [141] investigated CO_2_ methanation under DBD plasma activation without external heating with two different reactor configurations, pseudo-adiabatic and adiabatic approaches, in order to discern between the role of plasma activation and that of the reaction exothermicity. A Ni–Ce catalyst supported on Al_2_O_3_ was loaded into the reactors. In the pseudo-adiabatic condition, plasma application and the reaction heat increased the reaction up to 150 °C, while in the adiabatic system the temperature rose up to 350 °C. In order to obtain the same conversion values, the adiabatic configuration needed an input power 10 W less than the pseudo-adiabatic configuration. Thus, the authors obtained that a good management of released heat coupled with the adoption of an insulating system helps to maximize the energy efficiency of the overall process.

A summary of the research article on CO_2_ methanation reported in sub-Section 7.2 is provided in Table 8.

#### Conclusions

Based on the experimental and numeric evaluation reported in the above-cited works on plasma-assisted CO_2_ methanation, it is possible to observe that nonthermal plasma is a promising technology to perform the reaction. Nevertheless, as the main reaction product in absence of a proper catalyst is CO, in order to have a selective and efficient process a hybrid plasma–catalytic system is specifically needed to perform the Sabatier reaction. Nickel has been widely recognized as a highly performing catalyst towards CO_2_ methanation both in thermal and plasma-assisted process and of course this represents a very positive aspect, as it leads to relatively cheap and available catalysts. Furthermore, the reported studies highlighted that in the plasma-assisted process, both plasma and the reaction exothermicity act as activator, resulting in an overall better efficiency of the process.

## 8. VOCs Abatement via NTP Technology

With the continuous decrease of the emissions of particulate matter, SO_x_ and NO_x_, the abatement of volatile organic compounds (VOCs) emitted from various industries is becoming increasingly important, due to their environmental issues. In fact, VOCs have high photochemical reactivity and react easily with NO_x_ to form ozone [142]. Moreover, they are key precursor of secondary organic aerosols, which are significant components of fine particulate matter [3,4,5], as well as they are dangerous for human health, due to their carcinogenicity, affecting the central nervous system, causing different diseases, including respiratory ones [142]. The reduction of the VOCs emissions one approach is the use of the so-called “add-on” equipment, which recover or destroy off–gas VOC pollutants; a second approach could be the change in processes and/or raw materials to reduce or destroy VOCs generation. Regarding the former, different techniques have been developed, including absorption, adsorption, thermal oxidation, catalytic oxidation, photocatalysis, NTP-assisted oxidation, membrane technology [143].

One of the drawbacks of NTPs for VOCs abatement is the potential formation of unwanted byproducts, due to the partial conversion of the initial compounds [144,145]. The increase of the discharge power can help in preventing this formation, but it results in an increased energy consumption. Hence, the use of a catalyst, allowing the reduction of the activation energy and the induction of alternative and more energy-efficient reaction routes, can be a useful way for overcome this disadvantage [146,147]. In an NTP-assisted VOC oxidation the placement of the catalyst is one of the parameters to consider; the two main configurations are named “in plasma catalysis” (IPC) and “post-plasma catalysis” (PPC). In the former, the catalyst is directly located into the NTP reactor, which can be filled with the catalyst either totally (as in a packed-bed or monolithic reactor) [148,149,150,151] or partially (as in the hybrid reactor) [152,153,154]. In the last decade, different reviews have been published regarding the combined use of NTP and catalysts in the abatement of the several VOCs categories; all the reviews have considered all the main features, including reactor configuration, catalysts typology, catalyst placement, discharge power, number of electrodes [9,142,143,144,155,156,157,158,159,160]. The analysis of these reviews evidenced the enormous interest of the scientific community towards the issue of the VOCs abatement by means of the catalytic NTP-assisted oxidation processes, especially by using packed bed or DBD reactors. The first consideration about the reactor is that it should result in (i) high VOCs removal efficiency, (ii) high mineralization rate and (iii) low formation of byproducts. Moreover, also the feeding gas properties must be considered since its properties in terms of initial concentration, flow rate and humidity may greatly influence the VOCs abatement. As mentioned, a lot or studies have been made, also devoted to the industrial application of the NTP technology, but at the present the presence of commercial applications is quite scarce. Therefore, further studies are still needed in order to deeply understand the mechanisms behind the interactions between plasma and VOCs, in particular regarding the discharge process and the degradation pathways of VOCs with or without a catalyst. Recently, the direct observation of the discharge behavior as well as the propagation of streamers on catalyst surface, has been possible by using fast imaging technique. This technique, combined with fluid modeling, allows to have more information on variation and distribution of the temperature and density of electrons during discharge, so giving deeper insights into the discharge features, including electric field enhancement in packed bed DBD reactors, plasma streamer propagation in packed bed DBD reactors and plasma streamer penetration in catalyst pores.

Regarding the VOCs decomposition mechanisms, a major part of the researchers argued from the information obtained by ex situ detection techniques. Anyway, the application of these techniques does not allow to fully investigate the physical and chemical processes underlying behind the VOCs decomposition, including the effect of a catalyst, due to the short survival time of reactive species such as radicals and electrons in plasma and the intermediates generated from VOCs. Therefore, recently direct in situ detection techniques combined to DFT (density functional theory) have been applied, so resulting in more data for better understanding the mechanism for VOCs decomposition. However, for the optimization of the whole system the theoretical studies must work in cooperation with practical applications, which through dedicated scale-up experiments could lead to real and effective industrial applications. One example of possible research area could be the investigation of the performance of catalytic NTP-assisted processes on mixed VOCs removal, since real exhaust gas contains many types of VOCs. In this case, a critical aspect is surely the optimal combination of the right catalyst position (IPC or PPC) and of its composition. In fact, several studies have demonstrated that high mineralization rates can be obtained in IPC systems, while PPC system is effective for the suppression of residual ozone. Therefore, the addition of a downstream catalyst to an IPC system may provide an effective solution for simultaneously reducing the VOCs emissions and ensuring a low residual ozone level. However, a single catalyst could not be able to effectively remove various types of VOCs simultaneously; so, a multistage arrangement with different catalysts may be a valid option to address this issue. Therefore, the investigation of multistage catalytic systems is crucial in terms of both residual ozone elimination and complex VOCs mixtures treatment.

## 9. Conclusions

Plasma science has attracted the interest of researchers in various disciplines since the 1990s. This continuously evolving field has spawned investigations into several applications, including industrial sterilization, pollution control, polymer science, food safety and biomedicine. nonthermal plasma (NTP) can promote the occurrence of chemical reactions in a lower operating temperature range, condition in which, in a conventional process, a catalyst is generally not active. The aim, when using NTP, is to selectively transfer electrical energy to the electrons, generating free radicals through collisions and promoting the desired chemical changes without spending energy in heating the system. Therefore, NTP can be used in various fields. As examples, NTP can be used in the CO_2_ utilization processes in which in combination with a properly chosen catalyst, the CO_2_ reforming or methanation reaction can occur, so obtaining in the latter case clean fuel CH_4_ by using CO_2_ as a raw material; the application of an external electric field may result in the conversion of complex macromolecular pollutants into small molecules safe and free of pollutants, such as in the case of volatile organic compound (VOC) decomposition; NTP can also purify exhaust gases, such as in the case of NO_x_ removal from exhaust gases and soot removal from diesel engine exhaust; NTP may also be used for ammonia production. The application of NTP to the reviewed fields did not give the same successful results, since it resulted very effective in pollutants abatement especially by using DBD reactors coupled to properly selected catalysts, while some doubts raised when it was applied to ammonia production. In fact, in this last case the different studies evidenced that a further optimization of the system is needed for having an NTP-assisted ammonia production competitive with the conventional one. Regarding the CO_2_ utilization processes, the reviewed studies indicated that NTP is a promising technology, but only if coupled to a proper catalyst in both CO_2_ reforming and methanation, in order to assure the best selectivity and efficiency of the processes.

## Figures and Tables

**Table 1:** Features of the different plasmas.

Low Temperature Plasma			High Temperature Plasma
Thermal Plasma T_e_ ≈T_i_ ≈T_g_ ~10^4^ °C n_e_ ~10^20^ m^−3^	Non Thermal Plasma T_i_ « T_e_ ~10^4^ °C n_e_ ≤ 10^20^ m^−3^ Warm Plasma T_g_ ~10^3^ °C	Cold Plasma T_g_ ~10^2^ °C	T_e_ ≈T_i_ > 10^7^ °C n_e_ ≥ 10^20^ m^−3^
example: arc plasma at normal pressure	example: gliding arc plasma	example: low temperature glow discharge	example: fusion plasma

T_e_—electron temperature; T_i_—ion temperature; T_g_—gas temperature; n_e_—electron density.

**Table 3:** Operative conditions and efficiency for SO_2_-removal over various catalysts and photocatalysts.

Catalysts	NTP Technology	Operative Conditions	SO_2_ Conversion (%)	Ref.
MnCu/TiO_2_	DBD–WESP	8 kHz, 10 kV and 200 W SED = 280 J L^−1^ SO_2_: 1000 or 20,000 mg·m^−3^ NO: 200 mg·m^−3^ 10% O_2_ and N_2_ balancing Reacting flow rate: 4 L min^−1^ Cleaning water flow rate: 3.6 L min^−1^ 1 atm, 25 °C	100	[48]
MnCu/TiO_2_	DBD–WESP	8 kHz, 10 kV and 200 W SED = 280 J L^−1^ SO_2_: 20,000 mg m^−3^ NO: 400 mg m^−3^ 10% O_2_ and N_2_ balancing Reacting flow rate: 4 L min^−1^ Cleaning water flow rate: 3.6 L min^−1^ 1 atm, 25 °C	77	[48]
Al_2_O_3_	DBD	10 W DBD plasma 1% SO_2_, 4% H_2_, N_2_ balance, flow rate = 100 mL min^−1^ at 150 °C, 1 atm	17	[51]
FeS/Al_2_O_3_	DBD	10 W DBD plasma 1% SO_2_, 4% H_2_, N_2_ balance, flow rate = 100 mL min^−1^ at 150 °C, 1 atm	31	[51]
ZnS/ Al_2_O_3_	DBD	10 W DBD plasma 1% SO_2_, 4% H_2_, N_2_ balance, flow rate = 100 mL min^−1^ at 150 °C, 1 atm	28	[51]
Al_2_O_3_	DBD	10 W DBD plasma 1% SO_2_, 4% CH_4_, N_2_ balance, flow rate = 100 mL min^−1^ at 150 °C, 1 atm	27	[51]
FeS/Al_2_O_3_	DBD	10 W DBD plasma 1% SO_2_, 4% CH_4_, N_2_ balance, flow rate = 100 mL min^−1^ at 150 °C, 1 atm	34	[51]
ZnS/Al_2_O_3_	DBD	10 W DBD plasma 1% SO_2_, 4% CH_4_, N_2_ balance, flow rate = 100 mL min^−1^ at 150 °C, 1 atm	35	[51]
TiO_2_	DBD	900 Hz, 7 kV, residence time = 1 s SO_2_ = 100 ppm, H_2_O = 4%, SO_2_/NH_3_ = 2 21% O_2_ and N_2_ balancing 1 atm, 25 °C	100	[52]
TiO_2_	DBD	900 Hz, 7 kV, residence time = 1 s SO_2_ = 600 ppm, H_2_O = 4%, SO_2_/NH_3_ = 2 21% O_2_ and N_2_ balancing 1 atm, 25 °C	16	[52]
TiO_2_	DBD	900 Hz, 9 kV, residence time = 1 s SO_2_ = 400 ppm, H_2_O = 4%, SO_2_/NH_3_ = 2 21% O_2_ and N_2_ balancing 1 atm, 25 °C	77	[52]
TiO_2_	DBD	100 Hz, 9 kV, residence time = 1 s SO_2_ = 400 ppm, H_2_O = 4%, SO_2_/NH_3_ = 2 21% O_2_ and N_2_ balancing 1 atm, 25 °C	9	[52]
TiO_2_	DBD	900 Hz, 9 kV, residence time = 1 s SO_2_ = 400 ppm, H_2_O = 4%, SO_2_/NH_3_ = 2 21% O_2_ and N_2_ balancing 1 atm, 25 °C	55	[52]
TiO_2_	DBD	900 Hz, 9 kV, residence time = 0.32 s SO_2_ = 400 ppm, H_2_O = 4%, SO_2_/NH_3_ = 2 21% O_2_ and N_2_ balancing 1 atm, 25 °C	18	[52]
TiO_2_ deposited via dip-coating	DBD	900 Hz, 9 kV, residence time = 1 s SO_2_ = 420 ppm 21% O_2_ and N_2_ balancing 1 atm, 25 °C	45	[53]
TiO_2_ deposited via PCVD (layer thickness 150 nm)	DBD	900 Hz, 9 kV, residence time = 1 s SO_2_ = 420 ppm 21% O_2_ and N_2_ balancing 1 atm, 25 °C	68	[53]
TiO_2_ deposited via PCVD (layer thickness 35 nm)	DBD	900 Hz, 11 kV, residence time = 1 s SO_2_ = 260 ppm 21% O_2_ and N_2_ balancing 1 atm, 25 °C	15	[54]
TiO_2_ deposited via PCVD (layer thickness 600 nm)	DBD	900 Hz, 11 kV, residence time = 1 s SO_2_ = 260 ppm 21% O_2_ and N_2_ balancing 1 atm, 25 °C	100	[54]
Zeolite without TiO_2_ coating	DBD	900 Hz, 12 kV, residence time = 1 s SO_2_ = 200 ppm 21% O_2_ and N_2_ balancing 1, 25 °C	31	[55]
Zeolite with TiO_2_ coating	DBD	900 Hz, 12 kV, residence time = 1 s SO_2_ = 200 ppm 21% O_2_ and N_2_ balancing 1 atm, 25 °C	58	[55]
γ-Al_2_O_3_	DBD	7 W DBD Plasma NO = 440 ppm, NO_2_ = 14 ppm, SO_2_ = 460 ppm 21% O_2_ and N_2_ balancing Reacting flow rate: 600 mL min^−1^	24	[56]
TiO_2_	PCP	12.5 kV SO_2_: 906 ppm (N_2_ basis); O_2_: 2.1%; H_2_O: 0.18%; H_2_O_2_: 0.055% Reacting flow rate: 1 L min^−1^ 1 atm, 25 °C	42	[44]

**Table 4:** Operative conditions and efficiency for H_2_S-removal over various catalysts.

Catalysts	NTP Technology	Operative Conditions	H_2_S Conversion (%)	Ref.
CdS/Al_2_O_3_	DBD	10 kHz, 6.12 eV/H_2_ 20% O_2_ in Ar Reacting flow rate: 30 mL min^−1^ 1 atm, 120 °C	90.9	[66]
ZnS/Al_2_O_3_	DBD	10 kHz, 6.12 eV/H_2_ 20% O_2_ in Ar Reacting flow rate: 30 mL min^−1^ 1 atm, 120 °C	82.9	[66]
Zn_0.4_Cd_0.6_S/Al_2_O_3_	DBD	10 kHz, 6.12 eV/H_2_ 20% O_2_ in Ar Reacting flow rate: 30 mL min^−1^ 1 atm, 120 °C	97.9	[66]
Zn_0.6_Cd_0.4_S/Al_2_O_3_	DBD	10 kHz, 6.12 eV/H_2_ 20% O_2_ in Ar Reacting flow rate: 30 mL min^−1^ 1 atm, 120 °C	100	[68]
Zn_0.2_Cd_0.8_S/Al_2_O_3_	DBD	10 kHz, 6.12 eV/H_2_ 20% O_2_ in Ar Reacting flow rate: 30 mL min^−1^ 1 atm, 120 °C	92.8	[68]
Zn_0.8_Cd_0.2_S/Al_2_O_3_	DBD	10 kHz, 6.12 eV/H_2_ 20% O_2_ in Ar Reacting flow rate: 30 mL min^−1^ 1 atm, 120 °C	84.9	[68]
Cr_0.20_–ZnS/Al_2_O_3_	DBD	10 kHz, 5.57 eV/H_2_ 20% O_2_ in Ar Reacting flow rate: 30 mL min^−1^ 1 atm, 120 °C	100	[69]
Cr_0.25_–ZnS/Al_2_O_3_	DBD	10 kHz, 5.57 eV/H_2_ 20% O_2_ in Ar Reacting flow rate: 30 mL min^−1^ 1 atm, 120 °C	89.7	[69]
Cr_0.15_–ZnS/Al_2_O_3_	DBD	10 kHz, 5.57 eV/H_2_ 20% O_2_ in Ar Reacting flow rate: 30 mL min^−1^ 1 atm, 120 °C	87.4	[69]
Cr_0.10_–ZnS/Al_2_O_3_	DBD	10 kHz, 5.57 eV/H_2_ 20% O_2_ in Ar Reacting flow rate: 30 mL min^−1^ 1 atm, 120 °C	81.8	[69]
1-wt% MoS_2_/Al_2_O_3_	DBD	10 kHz, 95 kJ·L^−1^ H_2_S/CO_2_ ratio = 20:15 Reacting flow rate: 35 mL min^−1^ 1 atm, 120 °C	94	[70]
5-wt% MoS_2_/Al_2_O_3_	DBD	10 kHz, 95 kJ·L^−1^ H_2_S/CO_2_ ratio = 20:15 Reacting flow rate: 35 mL min^−1^ 1 atm, 120 °C	99	[70]
10-wt% MoS_2_/Al_2_O_3_	DBD	10 kHz, 95 kJ·L^−1^ H_2_S/CO_2_ ratio = 20:15 Reacting flow rate: 35 mL min^−1^ 1 atm, 120 °C	97	[70]
15-wt% MoS_2_/Al_2_O_3_	DBD	10 kHz, 95 kJ·L^−1^ H_2_S/CO_2_ ratio = 20:15 Reacting flow rate: 35 mL·min^−1^ 1 atm, 120 °C	92	[70]
3-wt% MoO_x_/Al_2_O_3_	DBD	10 kHz, 1 W 5% H_2_S/Ar Reacting flow rate: 150 mL min^−1^ 1 atm, 160 °C	48	[71]
5-wt% MoO_x_/Al_2_O_3_	DBD	10 kHz, 1 W 5% H_2_S/Ar Reacting flow rate: 150 mL min^−1^ 1 atm, 160 °C	52	[71]
7-wt% MoO_x_/Al_2_O_3_	DBD	10 kHz, 1 W 5% H_2_S/Ar Reacting flow rate: 150 mL min^−1^ 1 atm, 160 °C	45	[71]
Fe/WSAC Treated for 10 min at 6.8 kV	DBD	7.8 kHz, 6.8 kV 500 ppm of H_2_S in N_2_ Reacting flow rate: 60 mL min^−1^ 1 atm, 60 °C	100 for 270 min	[72]
Fe/WSAC Treated for 10 min at 6.8 kV with a gas gap of 5.5 mm and a dielectric thickness of 1.5 mm	DBD	7.8 kHz, 6.8 kV 500 ppm of H2S in N_2_ Reacting flow rate: 60 mL min^−1^ 1 atm, 60 °C	100 for 210 min	[73]
La_0.9_MnO_3_	DBD	10 kHz, 593.7 J·L^−1^ 100 ppm of H_2_S in air Reacting flow rate: 2 L min^−1^ 1 atm, 80 °C	96.4	[74]
Mn_2_O_3_	DBD	50 Hz, 22 kV 200 mg m^−3^ H_2_S, 1200-mg·m^−3^ O_3_ in air Reacting flow rate: 0.2 m^3^ h^−1^	100	[75]
Ag_2_O	DBD	50 Hz, 22 kV 200 mg m^−3^ H_2_S, 1200-mg m^−3^ O_3_ in air Reacting flow rate: 0.2 m^3^ h^−1^	98	[75]
CuO	DBD	50 Hz, 22 kV 200 mg m^−3^ H_2_S, 1200-mg·m^−3^ O_3_ in air Reacting flow rate: 0.2 m^3^ h^−1^	82	[75]
Fe_2_O_3_	DBD	50 Hz, 22 kV 200 mg m^−3^ H_2_S, 1200-mg m^−3^ O_3_ in air Reacting flow rate: 0.2 m^3^ h^−1^	75	[75]

**Table 5:** Operative conditions and efficiency for NO_x_-removal via NTP technology.

**Catalytic Removal via NTP Technology**				
**Catalyst Formulation**	**NTP Technology**	**Operative Conditions**	**NO_x_ Removal Efficiency %** **(Maximum Value Reached)**	**Reference**
Ag/Al_2_O_3_	DBD	16–23 kHz, 1–2 W Reacting flow rate: 276 cm^3^ min^−1^ NO: 720 ppm Either 540 ppm *n*-C_8_H_18_ or 620 ppm toluene 4.3% O_2_ 7.2% H_2_O 7.2% CO_2_ He as carrier gas 1 atm, 25–250 °C	70	[80]
Ag/α-Al_2_O_3_	DBD	2 W Reacting flow rate: 2 L min^−1^ NO: 300 ppm 10% O_2_ 3.2% H_2_O 265 ppm *n*-heptane N_2_ as balance gas	74	[81]
H–MOR Co–MOR NMOR	DBD	5 W Reacting flow rate: 60 mL min^−1^ NO: 2130 ppm 8% O_2_ He as balance gas T = 35 °C	99.6	[82]
Co/Ba/Al Pd/Co/Ba/Al Pd particle size = 3.1 nm Pt/Ba/Al Pt particle size = 2.7 nm	DBD	40 kHz, 20 W (in the rich phase) NO: 500 ppm 8% O_2_ 2% H_2_O 2% CO_2_ N_2_ as balance gas T = 150–350 °C	90	[83]
M/Ba/Al (M = Mn, Fe, Co, Ni and Cu)	DBD	0–40 kV, 40 kHz, 1.8 W (in the rich phase) phase) NO: 500 ppm 8% O_2_ 2% H_2_O Ar as balance gas T = 200–350 °C	100	[84]
Pt/Ba/Al Pt particle size = 2.7 nm Pd/Co/Ba/Al Pd particle size = 3.1 nm Pd/Ba/Al Pd particle size = 3.5 nm	DBD	40 kHz, 1.8 W (in the rich phase) NO: 500 ppm 8% O_2_ 2% H_2_O Ar as balance gas T = 150–350 °C	99	[85]
Pt/Ba/Al Pt particle size = 2.1 nm Pt/Co/Ba/Al Pt particle size = 1.6 nm Pt/Mn/Ba/Al Pt particle size = 1.8 nm Pt/Cu/Ba/Al Pt particle size = 2.1 nm	DBD	40 kHz, 1.8 W (in the rich phase) NO: 500 ppm 8% O_2_ 2% H_2_O Ar as balance gas T = 150–350 °C	80	[86]
Zeolites (H–ZSM-5)	DBD	0.03–4 W Reacting flow rate: 66 mL min^−1^ NO: 1800 ppm 10% O_2_ He as balance gas 1 atm, 25 °C	97.8	[87]
Cu-modified CMS	DBD	7.6 kV, 8.9 kHz, 200 W Reacting flow rate: 300 mL min^−1^ 0.05% NO 3% O_2_ N_2_ as balance gas	96.2	[88]
In/H–BEA zeolite In particle size = 6–10 nm	DBD	0–1.125 W Reacting flow rate: 500 mL min^−1^ NO: 440 ppm NO_2_: 14 ppm CH_4_: 600 ppm SO_2_: 0–100 ppm 6% O_2_ 7% H_2_O Ar as balance gas T = 230–570 °C	99	[89]
MnCu/TiO_2_	DBD–WESP	8 kHz, 10 kV and 200 W SO_2_: 0–2000 mg·m^−3^ NO: 200–400 mg·m^−3^ 10% O_2_ and N_2_ balancing Reacting flow rate: 4 L min^−1^ Cleaning water flow rate: 3.6 L min^−1^ 1 atm, 25 °C	93.4	[48]
γ-Al_2_O_3_	DBD	7 W NO: 440 ppm NO_2_: 14 ppm SO_2_: 460 ppm 21% O_2_ N_2_ balancing Reacting flow rate: 600 mL min^−1^ 1 atm, 25 °C	45	[56]
**Non-Catalytic Removal Via NTP Technology**				
**NTP Technology**	**Operative Conditions**		**NO_x_ Removal Efficiency %** **(Maximum Value Reached)**	**Reference**
DBD	0–7 kV, 1–2000 Hz		–	[90]
Volume barrier discharge (VBD)	80–500 Hz, 0.56 kW air 30 L min^−1^ Methane 10 L min^−1^		90	[91]
DBD	40 kV, 50 Hz NO: 100 ppm SO_2_: 300 ppm N_2_ as balance gas		80	[92]
DBD	16 kHz, 40 kV Reacting flow rate: 1 L min^−1^ NO: 300 ppm 5% O_2_ N_2_ as balance gas		77	[93]
DBD	60 kV, 5–25 kHz Reacting flow rate: 10 L min^−1^ NO: 500 ppm C_2_H_2_: 1000 ppm 6% O_2_ N_2_ as balance gas		40	[94]
DBD	0–5 kV, 15 kHz, 45 W Reacting flow rate: 1 L s^−1^ HC: 10 ppm NO_x_: 116 ppm 3.32% CO_2_ 0.5% CO 17.03% O_2_		95	[95]
DBD	23 kV, 20 kHz, 250 W Reacting flow rate: 150 L min^−1^ NO: 350 ppmv SO_2_: 800 ppmv		88.8	[96]
DBD	0–7 kV, 60 Hz NO: 300 ppm SO_2_: 0–1000 ppm 4.2% O_2_ N_2_ as balance gas		100	[97]

**Table 6:** Operative conditions and soot-removal efficiency via NTP technology.

Catalyst Formulation (Particle Size [nm])	NTP Technology	Operative Conditions	Soot-Removal Efficiency [g·kWh^−1^] (Maximum Value Reached)	Reference
Fe_2_O_3_ (14.6) MnO_x_ (168.1) Co_3_O_4_ (53.3)	Corona plasma reactor	0–20 kV, 4–7.5 W 300 sccm of feed gas containing 10% O_2_ in N_2_ Electrode surface coated with 3.5 mg of soot Residence time: 0.94 s 1 atm, 180–350 °C	7.0 with MnO_x_	[102]
Au, Pt, Pd and Ag (not specified)	DBD	5–6 kV, 4.5 W Reacting flow rate: 1 L min^−1^ 20% O_2_ 20 mg of PM dispersed in 2 mL of liquid ethanol, uniformly loaded on the alumina plate surface t = 1 h 1 atm, 100–250 °C	6.1 with Au	[104]
AgCe–C (Ag crystallite size < 3 nm, CeO_2_ crystallite size = 25.1 nm) AgCe–R (Ag crystallite size < 3 nm, CeO_2_ crystallite size = 14.8 nm)	O_3_ activation	O_3_/air flow = 1 L/min O_3_ concentration of 400 ppm T = 200 °C	Not specified	[105]
MgSO_4_ K_2_SO_4_ CaSO_4_·2H_2_O (not specified)	DBD	7.4–8 kV, 4.5 W 800 mL·min^−1^ of N_2_ 200 mL·min^−1^ of O_2_ 10 mg of PM dispersed in 1-mL aqueous ethanol, mixed with a 500-μL aqueous solution of metal sulfate T = 100–250 °C	3.8 with K_2_SO_4_	[106]
Ag/α-Al_2_O_3_ (not specified)	DBD	Reacting flow rate: 2 L min^−1^ 300 ppm of NO 265 ppm of n-heptane 48 ppm of naphthalene (soot simulant) 10% O_2_ 3.7% H_2_O N_2_ as balance gas T = 150–350 °C	Not specified	[107]

**Table 7:** NTP-assisted CO_2_ reforming of methane.

Catalyst Formulation (Metal Particle Size *)	NTP Technology	Operative Conditions	Ref.
TiO_2_/g–C_3_N_4_	DBD	4–12 kHz, 20–60 W Feed flowrate = 50 mL min^−1^ CO_2_:CH_4_ = 6:1 to 1:6	[115]
Ni/γAl_2_O_3_–MgO (12 nm)	DBD	1–30 kV, 7.5 kHz, 100 W Feed flowrate = 20 mL min^−1^ CH_4_:CO_2_ = 1	[116]
Ni/La_2_O_3_–MgAl_2_O_4_ (10.77 nm)	DBD	1–30 kV, 7.5 kHz, 100 W Feed flowrate = 20 mL min^−1^ CH_4_:CO_2_ = 1 T = 350 °C	[117]
Ni/La_2_O_3_–MgAl_2_O_4_	DBD	1–30 kV, 7.5 kHz, 33–116 W Feed flowrate = 20 mL min^−1^ CH_4_:CO_2_ = 1	[118]
Ni/Al_2_O_3_ (<6 nm)	DBD	12–26 kV, 50 Hz, 1.4–4.8 W CH_4_: 10% _vol_ in Ar balance CO_2_: 5% _vol_ in Ar balance Feed flowrate = 40 mL min^−1^ CH_4_:CO_2_ = 1: 2, 1:1, 2:1	[119]
BZT; BFN; glass beads	DBD	12.1–13.6 kV, 20 kHz Feed flowrate = 40 mL min^−1^ CH_4_:CO_2_ = 1	[120]
Ni/γ-Al_2_O_3_	DBD	30 kV, 10 kHz, 20–60 W Feed flowrate = 25–125 mL min^−1^ CH_4_:CO_2_ = 1	[121]
La_2_O_3_/alumina balls	DBD	24 kV, 800 Hz, 8 W Feed flowrate = 40 mL min^−1^ CO_2_:CH_4_ = 0.4, 1, 2.3 He dilution: 33.3%, 50%, 75%	[122]
Ni/AC	DBD	30 kV, 5–12 kHz, 45 W Feed flowrate = 50 mL min^−1^ CH_4_:CO_2_ = 1 T = 270 °C	[123]
Ni/α-Al_2_O_3_	DBD	15 kV, 5–30 kHz Feed flowrate = 100 mL min^−1^ CH_4_:CO_2_ = 1	[124]
NiCe_x_C (23.7–31.1 nm)	DBD	8.5 kHz, 40 W CH_4_:CO_2_ = 3:1 to 1:3 Feed flowrate = 50 mL min^−1^ CH_4_:CO_2_ = 1 T = 260 °C	[125]
ZrO_2_; UiO-67; 2% PtNP@UiO-67 (1–4 nm)	DBD	6–10 kV, 30 kHz, 11 W CH_4_ up to 5000 ppm in Ar balance CO_2_ up to 5000 ppm in Ar balance Feed flowrate = 100 mL min^−1^ CH_4_:CO_2_ = 0.5–1.5	[126]
LaNiO_3_@SiO_2_ NP (40 nm)	DBD	30 kV, 5–100 kHz, 50–200 W Feed flowrate = 50 mL min^−1^ CH_4_:CO_2_ = 1	[128]
CaO; CaCO_3_; Ca(OH)_2_	DBD	13.5 kV, 800 Hz, 8 W Feed flowrate = 40 mL min^−1^ CH_4_:CO_2_ = 2 T = room T; 100 °C	[130]
Pt–Sn/Al_2_O_3_; BaTiO_3_; HZSM-5	DDBD	Generator: CTP-2000 K (9 kHz) Power: 8.1–65.8 W	[131]
–	Gliding arc discharge	Frequency: 50 Hz	[132]
–	RGA	0.74–1.50 A Feed flowrate = 3.7; 4.7; 6.7 SLPM CO_2_:CH_4_ = 1; 1.5; 2 T_pre-heating_ = room T; 200 °C	[133]

**Table 8:** NTP-assisted catalytic CO_2_ methanation.

Catalyst Formulation (Metal Particle Size [nm])	NTP Technology	Operative Conditions	Ref.
Ni/γ-Al_2_O_3_ (14 nm)	DBD	12–29 kV, 100 Hz, 3.6 W Feed flowrate = 640 mL min^−1^ CO_2_:H_2_ = 1:4 T range = 100–400 °C	[137]
Ni/γ-Al_2_O_3_ (<4.3 nm)	DBD	10 kV, 52–55 kHz, 15–18 W Feed flowrate = 50 mL min^−1^ CO_2_:H_2_ = 1:4 T range = 150–400 °C	[138]
Ni/Ce_0.58_Zr_0.42_O_2_	DBD	15–19 kV, 41 kHz, 4–16 W Feed flowrate = 20–350 mL min^−1^ CO_2_:H_2_ = 1:4 T range = 130–255 °C	[139]
Ni/MOFs	DBD	6.0–7.5 kV, 20.3 kHz CO_2_:H_2_ = 1:4	[140]
Ni–CeO_2_/Al_2_O_3_	DBD	4–9 kV, 52 kHz, 5–25 W Feed flowrate = 200–1000 mL min^−1^ CO_2_:H_2_ = 1:4	[141]

## Acronyms

AC	activated carbon
BET	Brunauer–Emmett–Teller
CCSU	carbon capture, storage and utilization
CMS	carbon molecular sieves
DBD	dielectric barrier discharge
DBDR	dielectric barrier discharge reactor
DDBD	double dielectric barrier discharge
DFT	density functional theory
DPF	diesel particulate filter
DRM	dry reforming of methane
EI	energy injection
GA	gliding arc
GD	glow discharge
GHSV	gas hourly space velocity
HC–SCR	hydrocarbon catalytic reduction
IPC	in-plasma catalysis
LNT	lean NOx trap
MOF	metal organic frameworks
MR	methane reforming
MW	microwave
NMOR	natural mordenite
NP	nanoparticles
NSR	NOx storage and reduction
NTP	non-thermal plasma
PCP	pulsed-corona plasma
PCVD	plasma chemical vapor deposition
PM	particulate matter
PPC	post-plasma catalysis
RF	radio frequency
RGA	rotating gliding arc
RPM	revolution per minute
SCR	selective catalytic reduction
SED	specific energy density
SEI	specific energy input
SEM	scanning electron microscopy
SNCR	selective noncatalytic reduction
SRE	soot-removal efficiency
TEM	transmission electron microscopy
TPD	temperature programmed desorption
VBD	volume barrier discharge
VOCs	volatile organic compounds
WSAC	walnut-shell activated carbon
XPS	X-ray photoelectron spectroscopy
